# Photosynthetic apparatus efficiency, phenolic acid profiling and pattern of chosen phytohormones in pseudometallophyte *Alyssum montanum*

**DOI:** 10.1038/s41598-021-83695-y

**Published:** 2021-02-18

**Authors:** Ewa Muszyńska, Krzysztof M. Tokarz, Michał Dziurka, Mateusz Labudda, Kinga Dziurka, Barbara Tokarz

**Affiliations:** 1 Department of Botany, Institute of Biology, Warsaw University of Life Sciences-SGGW, Nowoursynowska 159, Building 37, 02-776 Warsaw, Poland; 2 Department of Botany, Physiology and Plant Protection, Faculty of Biotechnology and Horticulture, University of Agriculture in Krakow, Al. 29 Listopada 54, 31-425 Kraków, Poland; 3 Department of Developmental Biology, The Franciszek Górski Institute of Plant Physiology, Polish Academy of Sciences, Niezapominajek 21, 30-239 Kraków, Poland; 4 Department of Biochemistry and Microbiology, Institute of Biology, Warsaw University of Life Sciences-SGGW, Nowoursynowska 159, Building 37, 02-776 Warsaw, Poland

**Keywords:** Biotechnology, Physiology, Plant sciences, Environmental sciences

## Abstract

The present study investigated the response of non-metallicolous (NM) and metallicolous (M) *Alyssum montanum* shoots cultured in vitro on a medium supplemented simultaneously with heavy metals (HMs) to identify mechanisms involved in alleviating metal-induced damage. Plant status in respect to photosynthetic apparatus efficiency was determined and linked with changes in biochemical composition of shoots, namely phenolic acids’ and stress-related phytohormones. Results showed the considerable inter-ecotype differences in (1) the photosynthetic pigments’ amount, (2) the functioning of membrane electron transporters as well as (3) the linear and alternative electron transport pathways, whose lower values were reported in NM than in M HM-treated culture. Photosynthetic apparatus protection in M specimens was assured by the activation of cinnamic acid synthesis (by phenylalanine ammonia lyase) and its further transformations to benzoic acid derivatives with high ability to counteract oxidative stress, that was accompanied by the overexpression of jasmonic acid stimulating antioxidant machinery. In turn, detrimental HM effects on NM shoots could result from the diminution of most phenolics’ accumulation, and only the content of coumarate (produced by bifunctional phenylalanine/tyrosine ammonia lyase) and rosmarinic acid increased. All these together with an enhanced concentration of abscisic acid might suggest that NM strategy to cope with HMs is based mostly on a restriction of metal movement with transpiration flow and their limited distribution in leaves. Summarizing, our findings for the first time point out the physiological and metabolic adaptation of pseudometallophyte *A. montanum* to adverse conditions.

## Introduction

Heavy metals (HMs) are one of the main abiotic stress factors that lead to multiple alterations in plant physiology and biochemistry, including water uptake, nutrient distribution, transpiration or nitrogen metabolism^1–3^. After exceeding the optimum threshold, accumulated HMs may also affect photosynthesis in many ways. In particular, they damage the chloroplast membrane functions and components of the electron transport chain in the reaction center of photosystem (PS) II and I as well as alter the energy transfer rate from light-harvesting chlorophyll *a*/*b* protein complex (LHC) to both PS^4,5^. Since PSII was found to be more susceptible to metallic ions compared with PSI, the inhibition of its photochemical activity may also result from the replacement of magnesium, calcium or manganese by HM cations either in the chlorophyll of LHCII or in the internal PSII antenna system and chlorophyll *a* dimmer in reaction center^6,7^. What is more, metals may restrain photosynthesis by a decline of the photosynthetic pigment content or relative proportions of chlorophyll *a* to *b* due to the inhibition of enzymes involved in their biosynthesis^8–11^. All these changes impair the light phase of photosynthesis, whereas HM-induced drop in the CO_2_ fixation can be strongly related to the reduction of ribulose bisphosphate carboxylase/oxygenase activity^1^. Further, HMs may act indirectly on the photosynthesis by decreasing stomata number and conductance, which limit carbon dioxide (CO_2_) assimilation and its intercellular concentration^2^. In addition, metallic ions accumulated in leaves as main photosynthetic organs may contribute to their premature senescence and consequently to the reduction of light absorption surface. Although in such circumstances the efficiency of photosynthesis decreases, changes in photosynthetic machinery manifested in general growth disturbances strictly depend not only on the HM dose and the duration of exposure but also on plant species.

Under the exposure to environmental stress factors, plants often accumulate an array of diverse metabolites. Among them, phenolic compounds have multiple functions in acclimation processes to an excessive amount of HMs, since they possess a high tendency to bind metallic ions via hydroxyl and carboxyl groups as well as to scavenge reactive oxygen species (ROS)^11–13^. Phenols are mainly synthesized by phenylpropanoid pathway, whose early metabolites are trans-cinnamic acid produced during deamination of L-phenylalanine catalyzed by phenylalanine ammonia-lyase (PAL), and/or *p-*coumaric acid obtained from L-tyrosine as a substrate for tyrosine ammonia-lyase (TAL) or via cinnamate hydroxylation. In the further chemical reactions of coumarate, simple phenylpropanoids, including sequentially caffeic, ferulic, and sinapic acids, are formed^14^. On the other hand, the degradation of the cinnamic acid side chain may result in the composition of benzoic acid, which in turn can undergo the same hydroxylation and methylation reactions as cinnamic and *p*-coumaric acids, giving analogous derivatives, such as *p*-hydroxybenzoic or protocatechuic acids^15^. Both cinnamic acid and the structurally related benzoic acid, together with their derivatives belong to a specific group of phenols called phenolic acids. These firstly mentioned compounds serve as a starting point for the biosynthesis of many other important molecules, like monolignols (coniferyl-, sinapyl- and *p*-coumaryl alcohols), which compose lignan and lignin units, flavonoids, or coumarins^16–18^. In turn, benzoic acid derivatives are known for their particularly strong antioxidant properties that are the more effective the more hydroxyl moieties in aromatic rings occur^13^. Therefore, gallic acid, with three hydroxyl groups, shows the highest antioxidant activity, followed by dihydroxybenzoic acids (with two hydroxyl groups) such as gentisic and protocatechuic acids, whereas salicylic and *p*‐hydroxybenzoic acids (with one hydroxyl group) have the lowest ability to ROS scavenger.

Survival in the presence of HMs depends on complex networks of various cellular processes to overcome the negative effects of stress. In the signal cascade involved in plant responses, phytohormones play key roles as coordinators of metabolic adjustment leading to improved protection against stressors and thus providing better growth plasticity^19^. The common reaction to various abiotic factors is a drop in the concentration of phytohormones considered as growth stimulants (i.e. cytokinins, auxins, and gibberellins), which is accompanied by an enhanced accumulation of phytohormones inhibiting cell elongation and division as well as accelerating senescence and dormancy (i.e. abscisic and salicylic acids, jasmonates)^20,21^. In recent years, great progress has been achieved toward explaining the roles of exogenously applied phytohormones in the mitigation of metal toxicity^19,22^. Despite many efforts held to elucidate the functions of both individual phytohormones and their interactions under HM treatment, there is limited information about their profiling and significance in metallophytes as tolerant representatives that are the best adapted by microevolutionary changes to grow in heavily contaminated areas^23,24^.

Zinc, lead and cadmium occur abundantly in soils of the Olkusz Ore-bearing Region located in Southern Poland, which is considered as one of the longest-exploited industrial areas in Europe, where ore deposits have been mining and processing since the Middle Ages^25^. Although many centuries of human activity have drastically affected the local vegetation and landscape, they have also contributed to the development of valuable HM-tolerant plant communities on the oldest, but still highly contaminated waste heaps. In the present study, *Alyssum montanum* as a representative species of such calamine flora has been selected to verify HM effects on physiological and biochemical properties of shoots cultured under fully-controlled in vitro conditions that were applied deliberately to exclude potential alterations in plant metabolism caused by changing environmental factors, e.g. light intensity, temperature or pathogens. In turn, the comparative assessment of HM effects between different ecotypes of the same species allows us to identify specific tolerance mechanisms involved in alleviating metal-induced damages. The contrasting ecotypes of *A. montanum* were previously proved to react differently on applied metals at the level of structural integrity, protein metabolism as well as ROS accumulation and functioning of antioxidant machinery^18,26^. Our findings revealed that the peroxidase-flavonoid system is an important element of *A. montanum* defense mechanism against metal and oxidative stresses. It prompted us to deeper insight into pathways of phenolic compounds’ synthesis and changes. Therefore, the present research is directed toward determining the possible survival strategy of *A. montanum* ecotypes in respect to photosynthetic apparatus efficiency and phytochemical composition of shoots exposed to zinc (Zn), lead (Pb) and cadmium (Cd) to better understand the role of phenolic acids and stress-related phytohormones in biochemical adaptation to adverse conditions.

## Materials and methods

### Plant material and experimental treatment

Plant material constituted the in vitro shoot cultures of two contrasting *Alyssum montanum* ecotypes that were obtained earlier from seeds of specimens representing non-metallicolous populations (described further as NM) in Pińczów, near Kielce (Poland) and metallicolous (M) calamine populations from Zn-Pb waste heap in Olkusz Ore-Bearing Region (Poland). The stock culture was propagated on an optimized basal medium as described by Muszyńska et al.^18^. In the main experiment, apical fragments of shoots were put on propagation medium enriched simultaneously with zinc, lead, and cadmium salts in concentrations reflected their contents in the natural environment of M ecotype, i.e., 714.3 μM ZnSO_4_, 3.0 μM Pb(NO_3_)_2_, and 16.4 μM CdCl_2_. Control culture were multiplicated on HM-free medium. For each ecotype and treatment five shoots were placed in each of six Erlenmeyer flasks (200 mL) filled with the respective media (50 mL). Cultures were maintained for 8 weeks, with subculture after 4 weeks, in a growth chamber at 24 °C, under a 16 h photoperiod (irradiance 80 μmol/m^2^/s). Cool white fluorescent lamps were used as a light source.

All microscopic, biochemical and physiological analyses were done in parallel on shoots cultivated simultaneously under the same standardized conditions to avoid differences in fresh/dry matter content not resulting from the culture cultivation in the presence of heavy metals.

### Metal localization by scanning electron microscopy (SEM) and energy-dispersive X-ray spectroscopy (EDX) analysis

SEM observations and element mapping analysis were conducted on high-pressure frozen leaves. Chosen fully expanded leaves were cross-sectioned in their middle length, mounted on cryo-holder, and then quickly transferred into the cryo-preparation chamber at − 140 °C. The samples were covered using platinum and observed by scanning electron microscope (Auriga 60, Zeiss) at − 140 °C. The mapping of heavy metals in vascular bundles, trichomes and epidermis was performed at 20 kV of acceleration voltage using EDX Oxford detector. Metal concentrations are expressed in percentage by weight (% wt.).

### Estimation of photosynthetic apparatus efficiency

*Photosynthetic pigment content.* One hundred milligrams of freeze-dried shoot samples were homogenized with 80% acetone in ice-cold conditions and centrifuged (15 min, 2500×*g*, 4 °C). Then, the absorbance of samples was recorded at 470, 646, and 663 nm with BioSpectrometer kinetic (Eppendorf, Hamburg, Germany). The content of chlorophyll *a*, chlorophyll *b*, and carotenoids was calculated according to Wellburn^27^ equations and expressed as mg/g fresh weight (FW) of the sample. Additionally, the ratio of chlorophyll *a* to *b* and the ratio of total chlorophylls to carotenoids were also calculated.

#### Chlorophyll a fluorescence

After 8 weeks of cultivation, the chlorophyll *a* fluorescence was measured on leaves from in vitro cultured shoots using Handy-PEA spectrofluorometer (Hansatech, King’s Lynn, UK) according to standard procedures. Ten fully developed leaves of the middle part of shoots from each treatment were dark adapted for 25 min. Then, the fluorescence was induced by red light: λ_max_ = 650 nm, 2000 μmol (quants)/ m^2^/s and recorded curves were analyzed using the fluorometer producer’s software (PEA-Plus). To visualize functional and structural changes of Photosystem II (PSII) selected parameters were calculated according to Jiang et al.^28^ and Kalaji et al.^29^:

F_0_: Minimum fluorescence, when all PSII reaction centers (RCs) are open.

F_M_: Maximum fluorescence, when all PSII reaction centers are closed.

Area: Total complementary area between the fluorescence induction curve and F_M_.

F_V_: Variable fluorescence.

F_V_/F_M_: Maximum quantum yield of PSII.

F_V_/F_0_: Efficiency of the oxygen-evolving complex on the donor side of the PSII.

V_J_: Relative variable fluorescence at 2 ms (J-step), that refers to the number of closed RCs relative to the total number of RCs.

V_I_: Relative variable fluorescence at 30 ms (I-step); that reflects the ability of PSI and its acceptors to oxidize reduced plastoquinone.

Sm: Normalized total complementary area above the OJIP transient (reflecting multiple-turnover Q_A_ reduction events) or total electron carriers per RC.

ABS/RC: Absorption flux per RC; that reflects the proportion between chlorophyll *a* molecule amounts in fluorescence-emitting antenna complexes and the active reaction centers.

TR_o_/RC: Trapped energy flux per RC at t = 0.

ET_o_/RC: Electron transport flux per RC at t = 0.

DI_o_/RC: Dissipated energy flux per RC at t = 0.

ABS/CS_o_: Absorption flux per CS at t = 0; represents the amount of photon energy absorbed by the antenna associated with active and inactive reaction centres of PSII and their relationship.

TR_o_/CS_o_: Trapped energy flux per CS at t = 0.

ET_o_/CS_o_: Electron transport flux per CS at t = 0.

DI_o_/CS_o_: Dissipated energy flux per CS at t = 0.

RC/CS_o_: Amount of active PSII RCs per CS at t = 0.

φ_Po_: Maximum quantum yield of primary photochemistry at t = 0*;* that indicates the probability of trapping the energy of absorbed photons by PSII reaction centers.

φ_Eo_: Quantum yield for the reduction of end acceptors of PSI per photon absorbed.

ψ_Eo_: Probability (at time 0) that trapped exciton moves an electron into the electron transport chain beyond Q_A_.

ρ_Ro_: Efficiency with which a trapped exciton can move an electron into the electron transport chain from Q_A‾_ to the PSI and electron acceptors.

δ_Ro_: Efficiency with which an electron can move from the reduced intersystem electron acceptors to the PSI end electron acceptors.

φ_Ro_: Quantum yield for the reduction of end acceptors of PSI per photon absorbed.

### Analysis of enzymes’ activity involved in phenolic acids biosynthesis

Leaf samples (100 mg) were homogenized with 1 ml of ice-cold extraction buffer (pH 8.5) containing 50 mM tris(hydroxymethyl)aminomethane (Tris)-HCl, 2 mM 2-mercaptoethanol, 2% polyvinylpyrrolidone and 0.1% Triton X-100. Homogenates were centrifuged (4 °C, 20 min, 16,000×*g*) and supernatants were collected. The activity of phenylalanine ammonia-lyase (PAL) was measured according to Hodgins^30^. The supernatants were mixed with reaction mixtures contained 2 mM l-phenylalanine in 150 mM Tris–HCl buffer (pH 8.5). The absorbance at 240 nm was recorded for 20 min with reads every 1 min. The PAL activity was expressed in arbitrary units. The one arbitrary unit of PAL activity was defined as 1 µmole formed trans-cinnamate after 1 min per gram of FW, and results were finally expressed per hour and a gram of FW. The activity of tyrosine ammonia-lyase (TAL) was measured according to Fritz et al.^31^. The supernatants were mixed with reaction mixtures contained 2 mM L-tyrosine in 150 mM Tris–HCl buffer (pH 8.5). The absorbance at 286 nm was recorded for 20 min with reads every 1 min. The TAL activity was expressed in arbitrary units. The one arbitrary unit of TAL activity was defined as 1 µmole formed *p*-coumarate after 1 min per gram of FW, and results were finally expressed per hour and a gram of FW. To ensure the credibility and accuracy of analyzes, the control reactions were prepared. The first control sample contained only enzymatic extract but without the addition of enzyme substrates, and the second one had only substrates but without enzymatic extracts. The other measurement conditions were the same as in the case of complete samples. All measurements were conducted at 30 °C in a UV-Star 96-well plate (Greiner, Monroe, NC, USA) on a Varioskan LUX Multimode Microplate Reader (Thermo Scientific, Waltham, MA, USA).

### Determination of phenolic acids

Free phenolic acids were estimated according to the modified method of Hura et al.^32^. Plant material was lyophilized and homogenized with zirconia beads in a mixing mill (MM400, Retch, Germany). Triple extraction to organic solvent (methanol/water/formic acid, MeOH/H_2_O/HCOOH, 14/4/1 v/v) was conducted, joined supernatants were evaporated under N_2_. The residue after evaporation was reconstituted in 50 µl of 50% MeOH in 1 M HCOOH and diluted to 1.2 ml with 1 M HCOOH before clean-up on SPE cartridges (Discovery DPA-6S, 1 ml, 50 mg, Supelco, Bellafonte, PA, USA). Cartridges were activated with 1 ml of MeOH followed by 1 ml of 1 M HCOOH. After that samples were applied and slowly aspirated then cartridges were flushed with 1 ml of 1 M HCOOH. Phenolics were washed out with 2 × 0.5 ml of 5% ammonia in 60% methanol in water. Then samples were evaporated under N_2_, reconstituted in 250 µl of methanol, and analyzed on Agilent Infinity 1260 system with a fluorescence detector, FLD, (Agilent, Waldbronn, Germany). Phenolics were separated on the Zorbax Eclipse Plus Phenyl-Hexyl 3.5 µm 3.0 mm × 100 mm column under a linear gradient of 2% formic acid aqueous solution versus methanol. Excitation (Ex) and emission (Em) wavelengths were dynamically changed to meet optimal parameters for individual acid. Technical details are given by Gołębiowska-Pikania et al.^33^. Chemicals were supplied by Sigma-Aldrich (Poznan, Poland).

### Determination of endogenous jasmonates, abscisic and salicylic acid

Stress-related phytohormones (ABA, JA, SA, and respective intermediates) were measured according to Dziurka et al.^34^, Hura et al.^35^ and Płażek et al.^36^ employing ultrahigh performance liquid chromatography coupled to tandem mass spectrometry (UHPLC-MS/MS). Lyophilized and pulverized samples were spiked with a heavy-labeled internal standard solution consist of [^2^H_4_]salicylic acid, [^2^H_6_]*cis*,*trans*-abscisic acid, [^2^H_5_]jasmonic acid, [^2^H_5_]benzoic acid and [^2^H_5_]dinor-12-oxo phytodienoic acid. Samples were extracted the same as for phenolic acids analysis, evaporated and reconstituted in 3% MeOH in 1 M HCOOH, and cleaned up on to hybrid SPE cartridges (BondElut, 30 mg, 1 ml, Agilent Technologies, Santa Clara, CA, USA). An Agilent Infinity 1260 coupled to 6410 Triple Quad LC/MS with ESI interface (Agilent Technologies) and AscentisExpres RP-Amide analytical column (2.7 μm, 2.1 mm × 150 mm; Supelco) was used for analyses. Compounds of interest were monitored in MRM (multiple reactions monitoring) mode in positive ionization (details are given by 35, 36). Agilent Masshunter Quantitative Analysis software (https://www.agilent.com/en/products/software-informatics/mass-spectrometry-software/data-analysis/quantitative-analysis) was used to control the LC–MS/MS system and for data analysis. Quantification was based on calibration curves for authentic standards taking into account recoveries of heavy-labelled internal standards. [^2^H_5_]jasmonic acid was supplied by CND Isotopes (Quebec, Canada), [^2^H_5_]dinor-12-oxo phytodienoic acid was supplied by Cayman Chem. Comp. (Ann Arbor, MI, USA); all other standards were from OlChemim (Olomouc, Czech Republic) at the highest available purity. All other chemicals were supplied by Sigma-Aldrich (Poznan, Poland).

### Statistical analysis

All data were subjected to two-way analysis of variance with ecotype and treatment as factors. The statistically significant differences between means were verified by Tukey’s test at P < 0.05 level. Statistica program (TIBCO, version 13.3, Software Inc., Palo Alto, CA, USA; https://docs.tibco.com/products/tibco-statistica-13-3-0) was used for the calculations. Results for the enzyme activity, phenolic acids, phytohormones and photosynthetic pigments contents were determined in three biological replications, whereas for the chlorophyll *a* fluorescence from 10 replicates (leaves) for particular treatments.

## Results

### Metal content and localization in Alyssum montanum leaves

**Figure 1:**
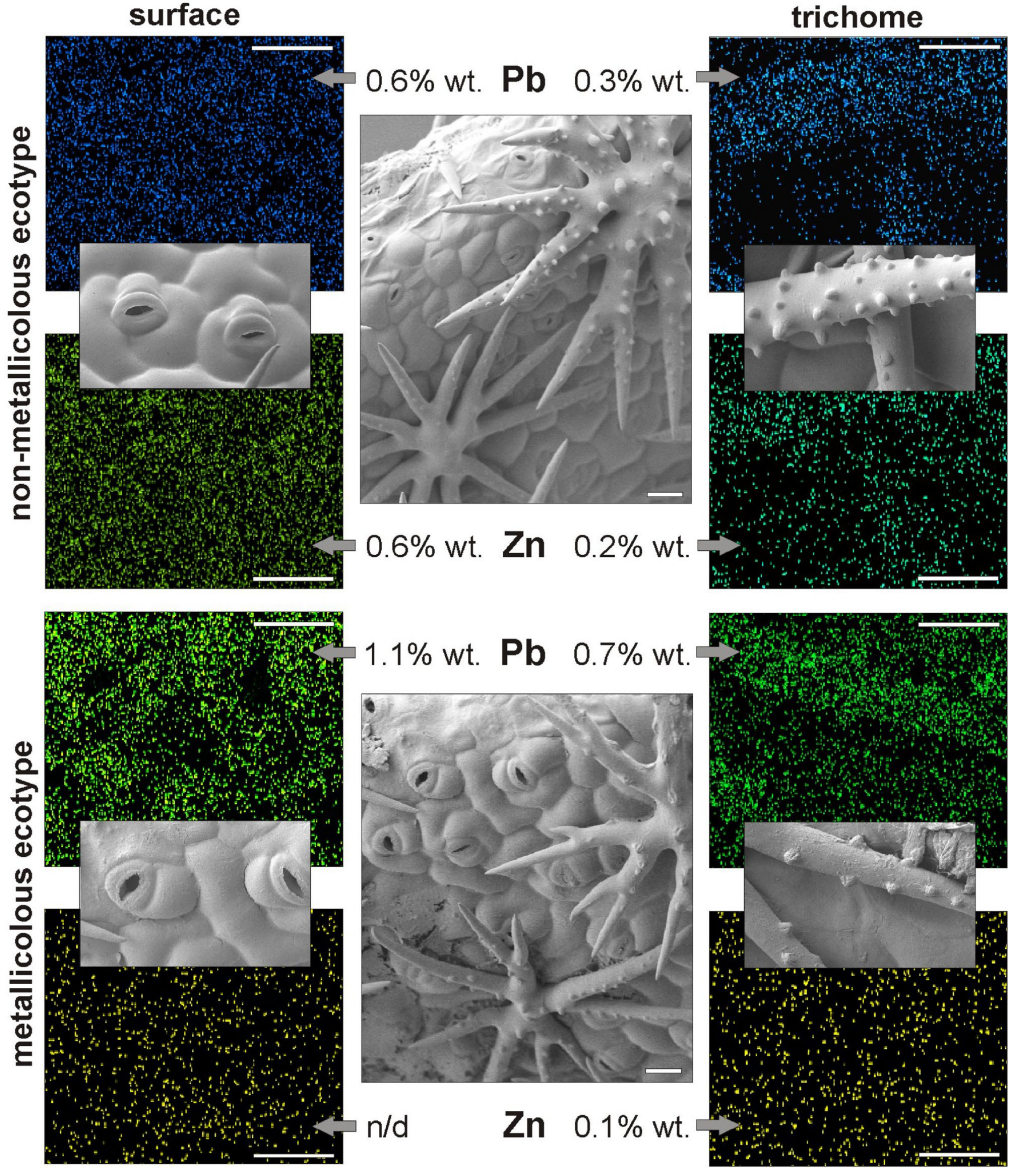
SEM–EDX micrographs of leaf surface and trichomes presenting lead (Pb) and zinc (Zn) distribution and content in contrasting *Alyssum montanum* ecotypes cultured on heavy metal-enriched medium. Bar 25 μm. *n/d* not detected.

During the experimental set, spontaneous rhizogenesis was almost not observed, and therefore shoots could be directly exposed to HMs (see Supplementary Fig. S1 online). The presence of metallic ions was noticed in both NM and M leaves, however, their amount and distribution differed between ecotypes (Fig. 1). It was found that the leaf surface of NM HM-treated culture accumulated similar content of Zn and Pb ions at the level of 0.6% wt. In turn, M leaves comprised 1.1% wt. of Pb, while Zn amount was below the detection level (Fig. 1). Both ecotypes exhibited comparable content of these metals in the main vascular bundle, which contained 0.1% and 0.4% wt. of Zn and Pb, respectively. Similarly, Cd accumulation at a low level of 0.1% wt. was observed regardless of ecotype and the above-mentioned tissues. Considering leaf trichomes, elemental mapping analysis revealed two times higher content of Zn in NM than M trichomes, in which 0.1% wt. was ascertained. Contrary, more than two-fold lower content of Pb was localized in NM trichomes (0.3% wt.) in comparison to M ones (0.7% wt.), whereas Cd ions were not detected at all (Fig. 1).

### Condition of photosynthetic apparatus in HM-treated specimens

Two-way analysis of variance revealed that the accumulation of chlorophyll *a* and the ratio of chlorophyll *a* to *b* were determined by all tested factors, chlorophyll *b* content together with total chlorophylls depended on ecotype and its combination with treatment, whereas carotenoids content and the calculated value of chlorophyll *a* + *b* to carotenoids depended separately on treatment and ecotype.

The toxicity of HMs to cultured NM shoots was manifested in a significant decrease of photosynthetic pigments, both chlorophylls and carotenoids, in comparison with control untreated culture (Table 1). Despite it, chlorophyll *a/b* ratio increased in shoots cultured on HM-containing medium, whereas the ratio of chlorophyll *a* + *b* to carotenoids remained at a similar level of 4.0–4.4 regardless of the treatment. In contrast, the application of HMs on M shoots resulted in enhanced accumulation of chlorophyll *a* and *b*, which in total reached approximately 0.9 mg/g FW and this value was about 16% higher than in control ones (Table 1). In turn, a statistically insignificant difference between both treatments was observed for carotenoid content that varied from 0.19 to 0.22 mg/g FW, however, a slight drop in this parameter was ascertained for HM-treated M shoots. Similarly, no influence of applied HMs was noticed on chlorophyll *a*/*b* ratio, while the ratio of total chlorophylls to carotenoids changed conversely and its increase to 4.9 in shoots from HM-containing medium was calculated.

**Table 1:** Photosynthetic pigments’ content and its ratios in *Alyssum montanum* shoots cultivated on control medium as well as in the presence of heavy metals (HMs).

Parameter	Non-metallicolous ecotype		Metallicolous ecotype	
	Control	HMs	Control	HMs
Chlorophyll *a* (mg/g FW)	0.591b*	0.374c	0.621b	0.706a
Chlorophyll *b* (mg/g FW)	0.172ab	0.099c	0.144b	0.203a
Chlorophyll *a*/*b*	3.445b	3.750a	3.508b	3.404b
Carotenoids (mg/g FW)	0.175b	0.118c	0.222a	0.188ab
Chlorophyll *a* + *b*/carotenoids	4.369ab	4.013bc	3.457c	4.851a

*Values are means of three replicates. Means indicated by the same letter do not significantly differ at P < 0.05 according to two-way ANOVA and post hoc Tukey’s test.

**Figure 2:**
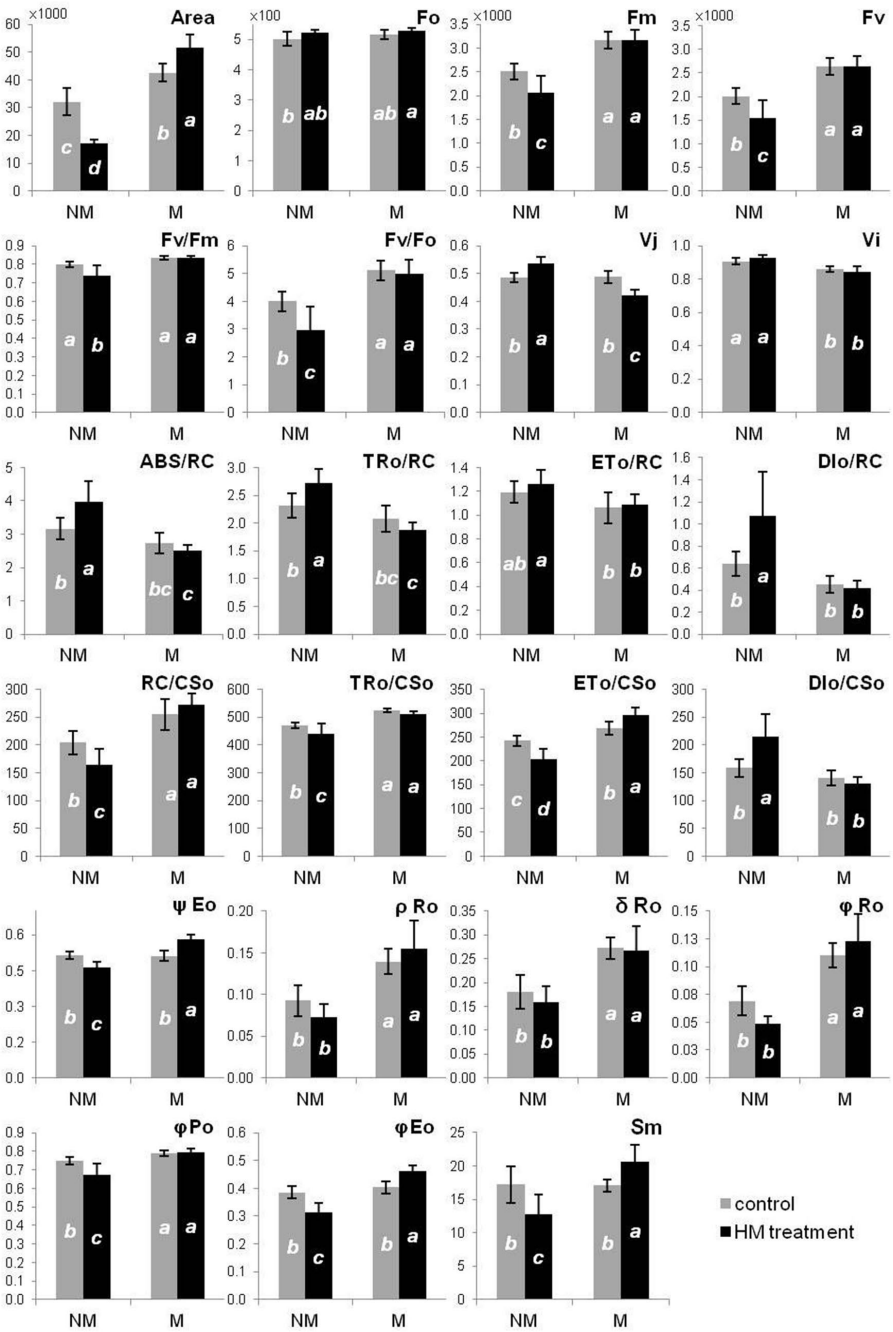
Comparison of raw PS II **s**tructural and functional parameters of *Alyssum montanum* leaves between non-metallicolous (NM) and metallicolous (M) ecotypes growing on control and heavy metal-enriched (HM) media. Different letters indicate statistically significant difference at P < 0.05 within each parameter according to two-way ANOVA and post hoc Tukey’s test. Abbreviations: *F*_*0*_ minimum fluorescence, when all PSII reaction centers (RCs) are open, *F*_*M*_ maximum fluorescence, when all PSII reaction centers are closed, *Area* total complementary area between the fluorescence induction curve and F_M_, *F*_*V*_ variable fluorescence, *F*_*V*_*/F*_*M*_ maximum quantum yield of PSII, *F*_*V*_*/F*_*0*_ efficiency of the oxygen-evolving complex on the donor side of the PSII, *V*_*J*_ relative variable fluorescence at 2 ms (J-step), that refers to the number of closed RCs relative to the total number of RCs, *V*_*I*_ relative variable fluorescence at 30 ms (I-step); that reflects the ability of PSI and its acceptors to oxidize reduced plastoquinone, *Sm* normalized total complementary area above the OJIP transient (reflecting multiple-turnover Q_A_ reduction events) or total electron carriers per RC, *ABS/RC* absorption flux per RC; that reflects the proportion between chlorophyll *a* molecule amounts in fluorescence-emitting antenna complexes and the active reaction centers, *TR*_*o*_*/RC* trapped energy flux per RC at t = 0, *ET*_*o*_*/RC* electron transport flux per RC at t = 0, *DI*_*o*_*/RC* dissipated energy flux per RC at t = 0, *ABS/CS*_*o*_ absorption flux per CS at t = 0; represents the amount of photon energy absorbed by the antenna associated with active and inactive reaction centres of PSII and their relationship, *TR*_*o*_*/CS*_*o*_ trapped energy flux per CS at t = 0, *ET*_*o*_*/CS*_*o*_ electron transport flux per CS at t = 0, *DI*_*o*_*/CS*_*o*_ dissipated energy flux per CS at t = 0, *RC/CS*_*o*_ amount of active PSII RCs per CS at t = 0, *φ*_*Po*_ maximum quantum yield of primary photochemistry at t = 0*;* that indicates the probability of trapping the energy of absorbed photons by PSII reaction centers, *φ*_*Eo*_ quantum yield for the reduction of end acceptors of PSI per photon absorbed, *ψ*_*Eo*_ probability (at time 0) that trapped exciton moves an electron into the electron transport chain beyond Q_A_, *ρ*_*Ro*_ efficiency with which a trapped exciton can move an electron into the electron transport chain from Q_A‾_ to the PSI and electron acceptors, *δ*_*Ro*_ efficiency with which an electron can move from the reduced intersystem electron acceptors to the PSI end electron acceptors, *φ*_*Ro*_ quantum yield for the reduction of end acceptors of PSI per photon absorbed.

Comparison of both ecotypes together unveiled differences between cultures growing on metal-free medium or with the addition of HMs (Fig. 2). Generally, M specimens were characterized by higher fluorescence parameters (F_M_, F_V_), plastoquinone pool (Area), the activity of oxygen-evolving complex (F_V_/F_0_), amount of active PSII reaction centers (RC) per cross-section (CS) (RC/CS_o_), specific fluxes per CS (TR_o_/SC_o_, ET_o_/CS_o_), electron flows parameters (ρR_o_, δR_o_) and quantum yield parameters (φP_o_, φR_o_) than NM plants both in control and HM treatment (Fig. 2). Besides, the maximum quantum yield of PSII (F_V_/F_M_), quantum yield for electron transport (φE_o_), probability of electron transport beyond Q_A-_ (ψE_o_) and total electron carriers per RC (Sm) increased in M shoots growing on medium enriched with HMs in comparison to NM ones (Fig. 2). In turn, V_j_ and V_i_ parameters, as well as specific fluxes per RC (ABS/RC, TR_o_/RC, ET_o_/RC, DI_o_/RC) and dissipation energy flux per CS (DI_o_/CS_o_) were significantly reduced in M specimens relative to NM ones under HM treatment (Fig. 2).

**Figure 3:**
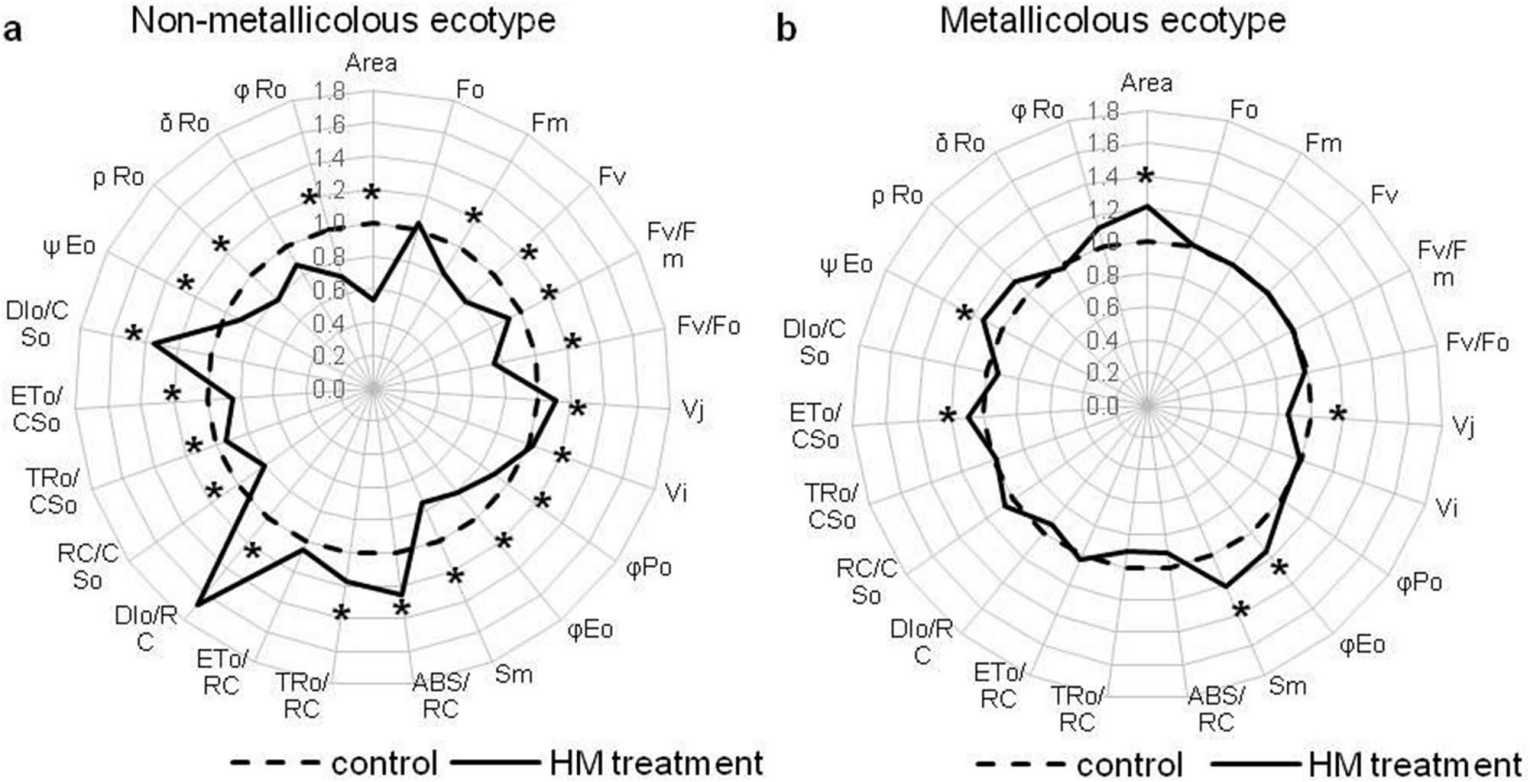
Structural and functional photosystem II parameters of *Alyssum montanum* leaves taken from non-metallicolous (**a**) and metallicolous (**b**) specimens, depending on treatment (control and heavy metals—HM); all the values were expressed relative to the control (set as 1); abbreviations—see Fig. 2; *statistically significant difference within each parameter in relation to control at  P < 0.05 according to post hoc Tukey’s test.

To reveal more detailed data on functional and structural changes in PSII after HM application, a separate comparison of each ecotype from control and HM-enriched medium was performed. Therefore, the values were normalized against the control value (= 1) and presented on the radar charts (Fig. 3a,b). In NM shoots treated with HMs, maximum (F_M_) and variable fluorescence (F_V_) as well as the maximum quantum yield of PSII (F_V_/F_M_) and activity of oxygen-evolving complex (F_V_/F_0_) declined in comparison to control ones (Fig. 3a). Moreover, plastoquinone pool (Area) and total electron carriers per RC (Sm) decreased in NM HM-treated shoots, whereas the number of closed RCs relative to the total number of RCs (V_j_) and a pool of rapidly reducing plastoquinone (V_i_) increased in these specimens (Fig. 3a). Furthermore, electron transport parameters (φE_o_, ψE_o_, ρR_o_) were reduced the same as quantum yields (φP_o_, φR_o_) (Fig. 3a). Then, specific fluxes per RC (ABS/RC, TR_o_/RC) increased while per CS (TR_o_/CS_o_, ET_o_/CS_o_) decreased after HM application on NM culture (Fig. 3a). In turn, the enhancement of dissipation energy flux both per RC (DI_o_/RC) and per CS (DI_o_/CS_o_) was observed in this treatment in comparison to control one (Fig. 3a). Whilst, in M ecotype considerably fewer differences in parameters of Photosystem II efficiency between untreated and HM-treated shoots, were noted (Fig. 3b). Statistically significant changes concerned Area and Sm parameters as well as φE_o_, ψE_o_ and ET_o_/CS_o_, whose increase was accompanied by V_j_ decrease in M culture from HM-containing medium (Fig. 3b).

### Activity of PAL and TAL in shoots after heavy metal application

The activity of two main enzymes involved in pathways of phenolic acid biosynthesis changed significantly in NM and M ecotypes, and this factor, as well as its combination with treatment were found to influence on both enzymes activity, whereas the tyrosine ammonia lyase (TAL) was additionally determined separately by the treatment.

In control NM specimens the phenylalanine ammonia lyase (PAL) activity was below the detection threshold, however, it increased about six times after HM application, and reached in NM HM-treated specimens about 5.89 U/g/h. In turn, M control culture exhibited a low level of PAL activity (0.75 U/g/h), which raised almost four times in HM-treated shoots (2.82 U/g/h). In this latter drop in the activity of tyrosine ammonia lyase (TAL) below detection level was ascertained, whereas in control M shoots it amounted 7.86 U/g/h. Interestingly, in NM shoots growing on HM-enriched medium the tendency was opposite and TAL activity increased up to 16.2 U/g/h and thus it was eight times higher than in control ones.

### Profile of phenolic acids in shoots from HM-containing medium

Most concentrations of the phenolic acid depended on ecotype, treatment and their combination except for protocatechuic and ferulic acids which dependent on ecotype and its combination with treatment, while the content of synapic and *p*-hydroxybenzoic acids was determined separately by ecotype and treatment.

In NM HM-treated shoots the content of the majority of analyzed phenolic acids decreased about 30–40% of untreated ones (Table 2). Statistically insignificant differences between control and exposed to HMs NM shoots were ascertained for cinnamic acid (19–20 µg/g DW), benzoic acid (0.75–0.77 µg/g DW) and *p*-hydroxybenzoic acid (0.32–0.35 µg/g DW), whereas the accumulation of rosmarinic, coumaric and homovanillic acids increased significantly in shoots growing in the presence of metallic ions. The opposite trend was observed for M specimens, in which the concentration of most phenolic acids was higher in HM-treated than untreated shoots (Table 2). Particularly important changes, that reached about twice of control values, were noticed for cinnamic, rosmarinic and protocatechuic acids. Additionally, the content of coumaric acid as well as p*-*hydroxybenzoic, gentisic and gallic acids increased in M shoots from HM-containing medium and amounted to 0.72 µg, 0.8 µg, 110 µg and 206 µg per g DW, respectively. In turn, control and treated M shoots accumulated similar level of ferulic, chlorogenic, homovanillic and vanillic acids. The detailed chromatograms of identified phenolic compounds in both NM and M plant samples can be found in Supplementary Figures S2–S4.

**Table 2:** Phenolic acids content and composition in shoots of two *Alyssum montanum* ecotypes cultured on control metal free-medium as well as medium enriched with heavy metals (HMs).

Phenolic compound (μg/g DW)	Non-metallicolous ecotype		Metallicolous ecotype	
	Control	HMs	Control	HMs
Cinnamic acid	18.97b*	19.54b	18.34b	38.98a
Rosmarinic acid	62.69b	70.02c	44.75d	104.24a
*p*-coumaric acid	91.15b	132.36a	54.18d	71.56c
Caffeic acid	660.36c	539.59d	1140.95a	910.91b
Ferulic acid	12.55b	10.43c	25.50a	26.22a
Sinapic acid	45.06a	32.49b	31. 70b	22.86c
Chlorogenic acid	80,984.18a	71,064.55b	5962.46c	5434.04c
Benzoic acid	0.75b	0.77b	1.00a	0.75b
*p*-hydroxybenzoic acid	0.32c	0.35c	0.55b	0.77a
Protocatechuic acid	18.71a	10.01b	5.55c	12.18b
Gentisic acid	60.19c	34.54d	88.90b	109.36a
Vanillic acid	13.79a	9.32b	1.99c	1.67c
Homovanillic acid	24.43b	38.07a	2.84c	3.14c
Syringic acid	160.19c	110.57d	410.10a	298.92b
Gallic acid	207.49a	131.26c	170.28b	205.89a

*Values are means of three replicates. Means indicated by the same letter do not significantly differ at P < 0.05 according to two-way ANOVA and post hoc Tukey’s test.

### Pattern of stress-related phytohormones in A. montanum shoots

Statistical analysis showed that the content of plant hormones was determined by both tested factors and their combination except for jasmonic and salicylic acids, which depended only on the combination of tested factors as well as separately by ecotype and treatment, respectively.

In NM shoots, the application of HMs led to significant increase in accumulation of both active abscisic acid and its inactive glucosyl ester, whereas the level of jasmonic acid and precursor of its synthesis (12-oxo-phytodienoic acid) as well as salicylic acid content was substantially reduced (Table 3; see also Supplementary Figs. S5, S6 online). In turn, M shoots growing in the presence of HMs were characterized by virtually the same level of abscisic and salicylic acids as in control untreated ones, which amounted to more than 200 and 104,000 ng/g DW, respectively (Table 3; see also Supplementary Figs. S5, S7 online). Despite it, an inactive ABA form was strongly accumulated in HM-treated specimens, and it reached about 35% more than in control ones. Among tested phytohormones, an increase in jasmonic acid content accompanied by a significant drop in 12-oxo-phytodienoic acid occurred as a reaction of M culture on HM exposure, although the concentration of this jasmonic precursor was generally lower in M than NM ecotype (Table 3). Interestingly, the content of jasmonic intermediate, that is jasmonic acid methyl ester, was not altered under HM treatment and regardless of ecotype it amounted from 7.9 to 8.4 ng/g DW (Table 3).

**Table 3:** Profile of chosen phytohormones in shoots of non-metallicolous (NM) and metallicolous (M) *Alyssum montanum* cultures growing on both control metal-free and heavy metal enriched (HMs) medium.

Treatment	Endogenous phytohormones (ng/g DW)					
	ABA	ABA-Glc	JA	12-OPDA	JA-Met	SA
NM control	335.99b	1129.65b	503.21c	68.01a	7.93a	127,445.11a
NM HMs	476.91a	3459.83a	401.72d	35.21b	8.07a	77,469.15b
M control	204.36c	658.82c	968.73b	4.06c	8.38a	103,934.58ab
M HMs	254.89c	1004.33b	1091.25a	1.63d	7.85a	102,524.31ab

Different letters indicate statistically significant means according to two-way ANOVA and post hoc Tukey’s test.

*ABA* abscisic acid, *ABA-Glc* abscisic acid glucosyl ester, *JA* jasmonic acid, *12-OPDA* 12-oxo-phytodienoic acid, *JA-Met* jasmonic acid methyl ester, *SA* salicylic acid.

## Discussion

Our previous experiment on *A. montanum* ecotypes showed that excess HM exposure elevated metal concentrations in shoots and roots to toxic levels^18^. The present experiment revealed more detailed information about HM localization and distribution in leaves as the main photosynthetic organs. Although Zn, Pb and Cd were detected at a similar level in vascular bundles of NM and M leaves, which indicated their analogous translocation in shoots, the further metal distribution quite differed. The comparable pattern of Zn and Pb presence in the whole NM leaf surface influenced probably physiological disturbances in these specimens. The negative response of NM culture could additionally be enhanced by the secretion of Zn, belonging to necessary trace element, in leaf trichomes, which resulted in its metabolic exclusion. In turn, trichomes of M specimens were used to detoxify an excess amount of harmful Pb, whose high accumulation was mostly detected here and in the whole leaf blade. These results particularize the histochemical method applied by us to general metallic ion visualization in *A. montanum* tissues^18^ and suggest that NM specimens do not exhibit any specific mechanism of HM elimination, whereas in M plants non-essential, potentially toxic elements can be partially removed from metabolically active central parts of the leaf blade to its outer surface.

### Metallic ions provoke ecotype-specific changes in photosynthetic apparatus efficiency

The photosynthetic apparatus, being responsible for the most important anabolic process of the plant—photosynthesis, is one of the most sensitive plant receptors. Therefore, the slightest imbalances in its redox state are important information for plants about alternations in the surrounding environment, leading to quick and effective acclimation in response to emerging biotic and abiotic stressors. Any redox disorders are associated with disruption of photosynthetically active radiation (PAR) energy flow starting from its perception place (Light Harvesting Complexes—LHCs) via photosystems (PSII, PSI) to its conversion in the form of stable reduced organic compounds (Calvin-Benson cycle). The condition of photosynthetic apparatus *A. montanum* ecotypes under multi-metal stress was evaluated for the first time. It was found that NM HM-treated shoots exhibited a noticeable reduction of photosynthetic pigment accumulation, which might result from disorders in chlorophyll synthesis, since tested HMs are known to inactivate the key enzyme—*δ*‐aminolevulinic acid dehydratase involved in this process, by permanently binding to its thiol (SH-) groups or indirectly via magnesium substitution at a catalytic site^8,37,38^. In addition, Pb may cause the reduction of carotenoid content by inactivation of enzymes in its synthesis pathway^5,9^ as well as induce degradation of chlorophyll *b*^39^. In this regard, our findings are in accordance with other studies on sensitive plant genotypes showing a significant drop in chlorophyll *a, b* and carotenoids content after the application of Pb^11,40^, Cd and Zn^6,41^. Contrary, plants resistant to Pb^11,38^, Zn and Cd^3,42^ were proved to have a constant level of photosynthetic pigments under high concentrations of these metals. Similarly, in M *A. montanum* shoots cultivated on a medium enriched with HMs, an increase of chlorophyll pigment content and no changes in carotenoid content was noted. It is noteworthy that the trend of changes in the photosynthetic pigment concentration in both M and NM shoots was the same, when pigments were calculated in relation to previously ascertained content of dry matter^18^. This may confirm that applied metals modified chlorophyll content regardless of tissue hydration.

An enhanced chlorophyll accumulation, especially chlorophyll *b*, is associated with the structural development of thylakoids^38^. As demonstrated by our previous study^18^, chloroplasts of the M ecotype were characterized by compact and undisturbed arrangements of granal thylakoids, whereas chloroplasts of NM plants showed ultrastructure disorders, manifested in the reduction of granal thylakoid, expansion of stromal thylakoids and numerous plastoglobules. Similar alternations of chloroplasts were observed in species susceptible either to Pb^5,11^, Zn^43^ and Cd^10^ applied separately or in the mixture^3^. Taking together, in NM specimens treated with HMs thylakoids destabilization under HM treatment could result from the damage of light harvesting complexes (LHCs), mainly LHCII, containing more than 50% of total plant chlorophylls^7^, whose reduced amount was ascertained in this ecotype. In turn, the enhanced accumulation of chlorophylls and unaffected ultrastructure of M chloroplasts indicate a highly effective mechanism of photosynthetic apparatus protection and acclimation to harsh conditions in these plants.

Metallic elements affect significantly photochemical and biochemical reactions in reductive photoconversion of CO_2_. Pb, Cd and Zn ions by binding to SH- groups of thylakoid membrane components modify membrane fluidity^5,7,38^. Furthermore, Pb and Cd significantly modify the amount of energy transferred from LHCII to RC PSII via structural disorders of membrane proteins building antenna systems^4^. Besides, on the PSII donor side, HMs negatively influence the functioning of the oxygen evolving-complex (OEC) and thus reduce the lumene acidification rate and electrons’ transport to RC PSII^44^. Such disruptions are reflected in altered parameters of chlorophyll *a* fluorescence^28,29^. The photosynthetic apparatus of plants susceptible to HMs demonstrates reduced efficiency of energy trapping, the lowered activity of OEC and increased dissipation of absorbed energy as heat^38,42,45^. Cultivation of NM specimens of *A. montanum* on HM-enriched medium resulted in a decrease in usage efficiency of trapped radiation (TR_o_/RC) whose quantity exceeded the photochemical efficiency of active RC PSII. It also inhibited the transfer of absorbed energy between supercomplexes (TR_o_/CS_o_) and intensified thermal energy dissipation (Di_o_/RC, Di_o_/CS_o_). Moreover, metallic ions caused a significant decrease in the activity of the OEC (F_v_/F_o_) in NM plants. In contrast, M plants treated with HMs were characterized by an effective transfer of absorbed energy to RC PSII (ψE_o_). Similar results were observed in species tolerant to toxic effects of Pb, Cd and Zn^38,42^.

A deleterious effect of Pb, Zn and Cd is often associated with disruptions of tertiary and quaternary RC PSII structures, membrane electron transporters (plastoquinone, plastocyanin, ferrodixins) and the whole group of thioredoxins, as well as enzymes involved in the biochemical phase of photosynthesis^5,38,42^. HM treatment significantly reduced the number of active RC PSII (V_j_ increase) and caused a significant decrease in their efficiency (φE_o_, ψE_o_) in NM plants. It is believed that decrease of RC efficiency results both from a decline of effective energy transfer on the PSII donor side in the closest vicinity of the PSII RC (V_i_ increase) and an electron transport efficiency decrease on the acceptor side of PSI (ρR_o_, δR_o_, φR_o_ decrease)^45,46^. Such changes were observed in NM specimens cultivated on HM-containing medium. A drop in the efficiency of the PSI donor side in connection with a reduction of both plastoquinone pool (Area) and other membrane electron transporters (Sm) indicates the lack of effective alternative electron flows, what lead to ROS generation and membrane lipid peroxidation as shown in the previous study^18^. Unlike, in M shoots cultivated on HM-enriched medium, a significant increase in the number of active RC PSII (V_j_ decrease) was observed together with their higher photochemical efficiency (ψE_o_). In addition, these specimens had a significantly higher content of all membrane electron transporters (Area, Sm). The observed functional adaptations of the photosynthetic apparatus and unaltered ultrastructure of chloroplasts^18^ suggest the possibility of a remarkably effective balance between linear and alternative electron transport pathways. The ability to quick electron redirection to alternative flows allows fast acclimation of the photosynthetic apparatus to the changed redox state in the presence of metallic elements. Moreover, the activation of secondary metabolites synthesis pathways, especially phenol derivatives, permits an efficient inactivation of ROS, and consequently, the protection of photosynthetic apparatus is assured.

### Phenolic acids’ biosynthesis and accumulation underlying the divergent response of Alyssum ecotypes to HMs

Phenolic compounds belong to secondary metabolites, which are not directly required for basic life processes, but they are proved to have a beneficial impact on plant survival in the hostile environments due to their protective roles against stress factors^13,14,47^. Accordingly, many studies revealed that various phenolic classes can be intensively produced under metal toxicity^3,12,14,48^, and therefore the modulation of their level and composition can be considered as a possible indicator of HM presence inside the protoplast. Despite it, minimal attention has been paid to both the overall phenolic profile of metallophytes and changes in these compounds formation after the application of metal doses corresponding to the natural growth condition of the metal-tolerant population. Our statistical analysis revealed that phenolic acids were determined by ecotype as well as its combination with treatment (except for synapic and *p*-hydroxybenzoic acids influenced separately by both applied factors) suggesting that this research field is particularly important to broaden the knowledge about acclimation and adaptation mechanisms to HMs.

The investigation of main enzymes involved in the pivotal step of phenols’ biosynthesis showed that the modification of their activity could affect the final phenolic acid content in *A. montanum* shoots and consequently modify plant response to metallic ions. In M specimens treated with HMs the increase in PAL activity positively correlated with the concentration of cinnamic acid, whose accumulation was significantly strengthened in comparison with control culture. Surprisingly, in NM shoots changes in cinnamate after metal applications were not detected although enhanced PAL activity was simultaneously ascertained. It might indicate that in NM ecotype monofunctional PAL producing trans-cinnamic acid was not identified, but instead bifunctional phenylalanine/tyrosine ammonia lyase (PTAL) turning over both amino acids could occur. Therefore, the level of *p-*coumarate as a reaction product of (P)TAL was the highest among all treatments in NM shoots cultivated on metal-enriched medium. Although PTAL is mainly restricted to grasses known from the unique composition of cell walls comprising mostly syringyl (S) units of lignin and cell wall-bound coumarates as well as from the possibility to generate nearly half of deposited lignin just from tyrosine, its overexpression can be also preferentially activated in other plants during stress-induced lignification^17,49^. In turn, (P)TAL activity in M HM-treated shoots was completely suppressed, confirming that cinnamate generation with the help of monofunctional PAL may be much more important in the successful alleviation of stress conditions. It can be related to the central position of cinnamic acid as a precursor in its further chemical conversions leading finally to the synthesis of both benzoic and *p*-coumaric acids and their hydroxylated derivatives with a wide spectrum of biological activities^15^. As an example, the most abundant hydroxycinnamic acid in plants called ferulic acid exhibits different functions including ROS scavenging, metal chelation and participation together with synapic and *p*-coumaric acids in the biosynthesis of lignan and lignin monomers^16,17^. Next, chlorogenic acid, an important ester of caffeic and quinic acids, protects against lipid peroxidation^50,51^. In the present experiment, the level of both ferulic and chlorogenic acids remained unchanged in *A. montanum* M shoots suggesting their independence on metal presence, constitutive mode of action*.* Contrary, the accumulation benzoic acid derivatives with high antioxidant ability was significantly stimulated in M ecotype under HM exposure. Taking into account their more pronounced fluctuations in comparison with derivatives of cinnamate in *A. montanum* M HM-treated shoots, it can be assumed that the synthesis of these phenolics may provide efficient photosynthetic apparatus protection as well as the explanation of less-marked changes in oxidative lipid peroxidation and protein carbonylation^18,26^. In turn, the content of the most of analyzed phenolic acids diminished significantly in NM shoots from HM-containing medium, and therefore symptoms of metal toxicity, such as photosynthetic apparatus disorders and oxidative stress resulting in morphological disturbances^18,26^ were observed, although the applied doses of metals did not ultimately lead to culture death.

Noteworthy is also the strongly enhanced accumulation of protocatechuate in HM-treated M shoots, which may contribute to effective metal detoxification since its role as metal chelators was earlier described, among others, in roots of *Matricaria chamomilla* exposed to Cu or Cd^47,52^ as well as in *Brassica juncea* treated with Cd^53^. The importance of hydroxyl benzoic acid derivatives in *A. montanum* tolerance to HMs could be significantly strengthened by rosmarinic acid (a caffeic acid ester) as well as *p*-coumaric acid, however, their increase seems to be a common reaction occurring in both NM and M specimens. Likewise, the synthesis of rosmarinic acid was comparably stimulated in control and Zn- or Pb-treated plants of *Echium vulgare* with different levels of metal tolerance^54^. In turn, the intensified accumulation of *p*-coumaric acid was observed for example in *Kandelia obovata* under Zn or Cd exposure^51^. Considering our previous research^18^, we can hypothesize that the further fate of *p-*coumarate might differ in both *A. montanum* ecotypes. In M specimens, which were characterized by an elevated content of flavonols, the activation of flavonoid pathway may lead to better and even more efficient detoxification of metal-induced ROS. Contrary, NM reaction to HMs was the opposite and a significant drop in both flavonols and anthocyanins concentration was determined^18^. Therefore, it is highly probable that in NM ecotype, *p*-coumarate could participate in the formation of other secondary structures limiting cell-to-cell penetration of metallic ions rather than ROS scavenging^47^. The examination of these potential component biosynthesis pathways and their role in NM response to HMs will be undertaken in the near future to support this preliminary hypothesis.

### Abscisic and jasmonic acids modulate Zn, Pb and Cd effects on Alyssum specimens

Plant ability to sustain life under stress pressure depends on several metabolic adaptations which are strictly regulated and integrated by the phytohormonal system. Among many research, information about endogenous hormonal balance in metallophytes is rather scarce, although the comparative assessment of changes in stress-related phytohormones between metal-tolerant and intolerant specimens could shed new light on their functional significance in tolerance to HMs. Our study on *A. montanum* showed an independent on ecotype comparable levels of jasmonic acid methyl ester (JA-Met) and salicylic acid (SA), albeit the biosynthesis of this latter decreased significantly in NM HM-treated shoots. Therefore, they can be recognized as components specific rather for species than for particular ecotypes that do not play an essential role in the adaptive response to harsh conditions. The positive relationship between endogenous SA content and the activity of glutathione peroxidase and glutathione *S*-transferase involved in glutathione transformations in control and HM-treated shoots of both ecotypes^18^, may suggest the constitutive role of SA as a regulator of the internal glutathione cycle. A similar observation was also made for *Thlaspi* species^23^ and *Triticum* varieties^55^ with different levels of HM tolerance that were exposed to Ni and Cd ions, respectively.

Modulatory effects of phytohormones on *A. montanum* response to HMs can be assigned to abscisic and jasmonic acids, whose enhanced accumulation distinguished particular ecotypes the most. In NM specimens the perception of HMs triggered the activation of ABA synthesis and its glucosyl ester, which constitutes an easily hydrolysable ABA conjugate. The endogenous level of ABA and its reservoir in plant tissues is known to increase mostly under drought, osmotic and salt stress^20,21^, although it was also found to change after HM exposure^56,57^. The mechanism of ABA protection against adverse conditions is associated with the regulation of plant water status by the adjustment of stomatal movement and thus transpiration rate^20^. It is possible that in NM *A. montanum* culture from HM-containing medium increased ABA concentration provoked the suppression of transpiration flow resulting in a limitation of metallic ions distribution in leaves. Such regulatory mechanism of restricted metal translocation could be activated just after exceeding the certain level of HM inside the mesophyll cell, and therefore the comparable Zn and Pb amount in xylem sap was still noticed in both ecotypes, but their further surface localization slightly differed between NM and M leaves. It cannot be also excluded that enhanced ABA accumulation in NM specimens confirmed HM-induced senescence events, especially if the loss of soluble proteins accompanied by the increase in proteolytic activity is taken into consideration^26^. In turn, M HM-treated shoots were characterized by stimulated production of jasmonic acid (JA), whereas ABA content remained constant in both treatments. Although these phytohormones share the common target of limiting water losses by stomatal closure^58^, JA is also known to ameliorate oxidative damage through the activation of antioxidant machinery^22,59,60^. In the case of our study, JA seemed to take part in the ROS removal pathways rather than in stomatal closure since this last process often refers to the restricted conversion of 12-OPDA to JA, which then promotes the stomatal movement individually or jointly to ABA^21^. Regarding M culture, a diminished level of jasmonate precursor was noticed under HM treatment, and therefore the role of JA in ROS scavenging could be supported. The ameliorative effects of JA on Ni-induced oxidative stress were also proved for serpentine and non-metalliferous *Alyssum inflatum* specimens, in which the reduction of ROS content occurred due to the enhancement accumulation of enzymatic and non-enzymatic antioxidants^24^. Further, Sirhindi et al.^60^ reported that JA supplementation minimized H_2_O_2_ accumulation in *Glycine max* under Ni stress through the improvement of superoxide dismutase, peroxidases and catalase activity. According to these findings, the mechanism responsible for HM tolerance in M ecotype may be related to the effective synthesis of JA as a signalling molecule that combats detrimental HMs effects by rapid ROS neutralization involving secondary metabolites as well as peroxidases and catalase, whose presence was previously visualized by histochemical method in leaves of HM-treated M culture^18^. Interestingly, M specimens growing on HM-enriched medium showed also strongly enhanced accumulation of ABA glucosyl ester, but free ABA level did not change in comparison to control. It may implicate the antagonistic relationship of ABA and JA in *A. montanum* response to HMs, and therefore inactive ABA form was not still hydrolyzed. Undoubtedly, research on the multifarious cross-talk of these plant hormones and other signal transduction pathways are worthy to be undertaken to better understand tolerance mechanisms in respect to reprogramming of metallophytes metabolism activated by long-term exposure to HMs.

**Figure 4:**
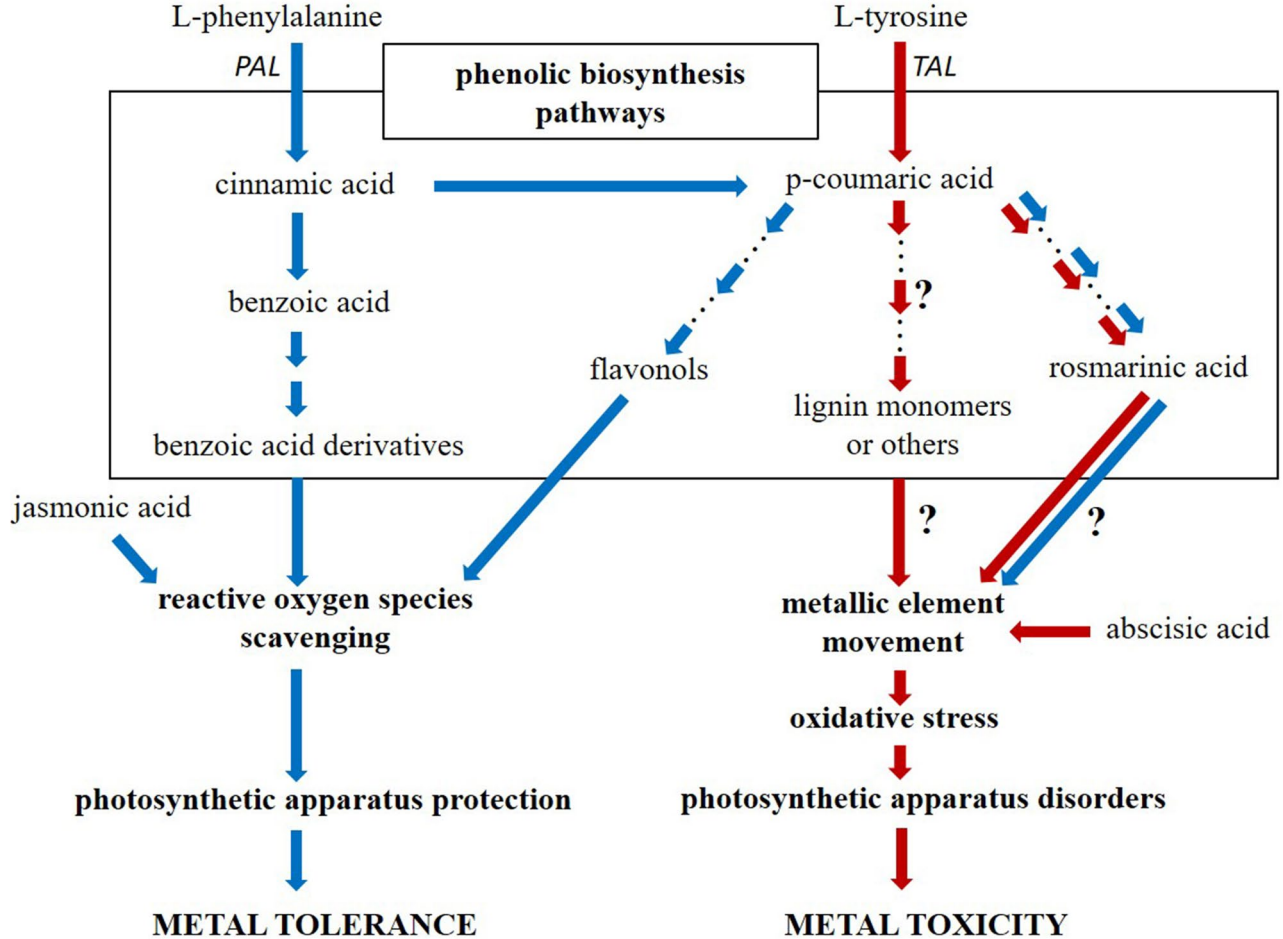
Schematic representation of physiological actions (bold font) leading to metal tolerance or toxicity, which result from the activation of ecotype-specific pathways of phenolic compound biosynthesis as well as different phytohormones operating in metallicolous and non-metallicolous *Alyssum montanum* specimens treated with Zn, Pb and Cd ions. Arrows indicate the successive stages of changes taking place in metallicolous (blue color) and non-metallicolous (red color) ecotype; arrow(s)–dots–arrow(s) suggest a long way of metabolic changes between particular compounds; question marks point to hypotheses to be tested by future work. This conceptual graph is based on the results from the present study and our previous experiments^18,26^ on both ecotypes.

The conceptual diagram comparing the metabolic responses of NM and M *A. montanum* ecotypes is shown in Fig. 4 as a summary of data collected so far both in the present and previous experiments^18,26^ as well as to identify paths of research that still represent a mystery worth to be exploring in the future.

## Conclusions

Metallicolous ecotype of *Alyssum montanum* can preserve their photosystems in two parallel ways. One of them relies on the effective protection of both the photosynthetic pigment synthesis pathway and the key structural elements of the photosynthetic apparatus. The second one is based on the rearrangement of electron transport pathways allowing for dynamic changes in the flow directions between linear transport and alternative pathways. The observed effects result from the increased synthesis of membrane electron transporters as well as from the enhanced accumulation of phenolic acids (mainly benzoic acid derivatives) providing an efficient neutralization of metal-induced ROS. This defence mechanism based on phenolics is accompanied by the exaggeration of jasmonic acid, which seems to take part in ROS removal pathways, too (Fig. 4). Simultaneously, M specimens eliminate toxic ions by their translocation from the metabolically active tissues of the leaf blade to its outer surface and trichomes. In turn, in the non-metallicolous, sensitive ecotype the destruction of photosystems, the destabilization of thylakoids and a decrease in chlorophyll content are observed together with the lack of effective electron flows leading to an uncontrolled ROS generation. Despite the increase in the activity of the (P)TAL enzyme, the content of most phenolic acids decreased which may suggest an ineffective ROS scavenging system. Thus, NM strategy to cope with an excess amount of HMs can rely on the reduced metal distribution associated with abscisic acid, which provokes stomatal closure and thereby limits metal uptake and translocation, rather than on the counteraction of oxidative stress (Fig. 4).

## Supplementary Information

Supplementary Information.

## References

[1] Per TS, Khan S, Asgher M, Bano B, Khan NA (2016). Photosynthetic and growth responses of two mustard cultivars differing in phytocystatin activity under cadmium stress. Photosynthetica.

[2] García-Sánchez, I.E., Barradasa, V.L., Ponce de León Hill, C.A., Esperón-Rodríguez, M., Pérez, I.R. & Ballinas, M. Effect of heavy metals and environmental variables on the assimilation of CO_2_ and stomatal conductance of *Ligustrum lucidum*, an urban tree from Mexico City. *Urban For Urban Gree.* **42**, 72–81 (2019).

[3] Muszyńska E, Labudda M, Kral A (2020). Ecotype-specific pathways of reactive oxygen species deactivation in facultative metallophyte *Silene vulgaris* (Moench) Garcke treated with heavy metals. Antioxidants.

[4] Dao LH, Beardall J (2016). Effects of lead on two green microalgae *Chlorella* and *Scenedesmus*: photosystem II activity and heterogeneity. Algal Res..

[5] Tokarz, K.M., Makowski, W., Tokarz, B., Hanula, M., Sitek, E., Muszyńska, E. et al. Can Ceylon Leadwort (*Plumbago zeylanica* L.) Acclimate to Lead Toxicity?—Studies of Photosynthetic Apparatus Efficiency. *Int. J. Mol. Sci.* **21(5)**, 1866 (2020a). 10.3390/ijms21051866 PMC7084747 32182862

[6] Küpper H, Parameswaran A, Leitenmaier B, Trtílek M, Šetlík I (2007). Cadmium-induced inhibition of photosynthesis and long-term acclimation to cadmium stress in the hyperaccumulator *Thlaspi caerulescens*. New Phytol..

[7] Tokarz, K., Piwowarczyk, B. & Makowski, W. Mechanisms involved in photosynthetic apparatus protection against lead toxicity in *Lead in Plants and the Environment. Radionuclides and Heavy Metals in the Environment* (eds. Gupta, D., Chatterjee, S., Walther, C.). 117–128 (Springer, Cham, Switzerland, 2020b).

[8] Cenkci S, Cigerci IH, Yildiz M, Özay C, Bozdag A, Terzi H (2010). Lead contamination reduces chlorophyll biosynthesis and genomic template stability in *Brassica rapa* L. Environ. Exp. Bot..

[9] Giuliano G (2014). Plant carotenoids: Genomics meets multi-gene engineering. Curr. Opin. Plant Biol..

[10] Figlioli F, Sorrentino MC, Memoli V, Arena C, Maisto G, Giordano S (2019). Overall plant responses to Cd and Pb metal stress in maize: Growth pattern, ultrastructure, and photosynthetic activity. Environ. Sci. Pollut. Res..

[11] Muszyńska E, Labudda M, Kamińska I, Górecka M, Bederska-Błaszczyk M (2019). Evaluation of heavy metal-induced responses in *Silene vulgaris* ecotypes. Protoplasma.

[12] Manquián-Cerda, K., Cruces, E., Escudey, M., Zúñiga, G. & Calderón, R. Interactive effects of aluminum and cadmium on phenolic compounds, antioxidant enzyme activity and oxidative stress in blueberry (*Vaccinium corymbosum* L.) plantlets cultivated *in vitro*. *Ecotox. Environ. Safe.* **150**, 320–326 (2018). 10.1016/j.ecoenv.2017.12.050 29294441

[13] Wang J, Yuan B, Huang B (2019). Differential heat-induced changes in phenolic acids associated with genotypic variations in heat tolerance for hard fescue. Crop Sci..

[14] Kisa D, Elmastaş M, Öztürk L, Kayιr Ö (2016). Responses of the phenolic compounds of *Zea mays* under heavy metal stress. Appl. Biol. Chem..

[15] Heleno SA, Martins A, Queiroz MJRP, Ferreira ICFR (2015). Bioactivity of phenolic acids: Metabolites versus parent compounds: A review. Food Chem..

[16] Kumar N, Pruthi V (2014). Potential applications of ferulic acid from natural sources. Biotechnol. Rep..

[17] Barros J, Serrani-Yarce J, Chen F, Baxter D, Venables BJ, Dixon RA (2016). Role of bifunctional ammonia-lyase in grass cell wall biosynthesis. Nature Plants.

[18] Muszyńska E, Labudda M, Różańska E, Hanus-Fajerska E, Znojek E (2018). Heavy metal tolerance in contrasting ecotypes of *Alyssum montanum*. Ecotox. Environ. Safe..

[19] Bücker-Neto L, Paiva ALS, Machado RD, Arenhart RA, Margis-Pinheiro M (2017). Interactions between plant hormones and heavy metals responses. Genet. Mol. Biol..

[20] Pantin F, Monnet F, Jannaud D, Costa JM, Renaud J, Muller B (2013). The dual effect of abscisic acid on stomata. New Phytol..

[21] Savchenko T, Kolla VA, Wang CQ, Nasafi Z, Hicks DR, Phadungchob B (2014). Functional convergence of oxylipin and abscisic acid pathways controls stomatal closure in response to drought. Plant Physiol..

[22] Wiszniewska A, Muszyńska E, Hanus-Fajerska E, Dziurka K, Dziurka M (2018). Evaluation of the protective role of exogenous growth regulators against Ni toxicity in woody shrub *Daphne jasmine*. Planta.

[23] Freeman JL, Garcia D, Kim D, Hopf A, Salt DE (2005). Constitutively elevated salicylic acid signals glutathione-mediated nickel tolerance in *Thlaspi* nickel hyperaccumulators. Plant Physiol..

[24] Kakavand, S.N, Karimi, N. & Ghasempour, H.-R. Salicylic acid and jasmonic acid restrains nickel toxicity by ameliorating antioxidant defense system in shoots of metallicolous and non-metallicolous *Alyssum inflatum* Náyr. populations. *Plant Physiol. Biochem.* **135**, 450–459 (2019). 10.1016/j.plaphy.2018.11.015 30497973

[25] Godzik, B. & Woch, M. History of mining in the Olkusz region in *Natural and historical values of Olkusz Ore-bearing Region* (ed. Godzik, B.) 29–36 (Drukarnia Kolejowa, Poland, 2015).

[26] Muszyńska E, Labudda M, Hanus-Fajerska E (2019). Changes in proteolytic activity and protein carbonylation in shoots of *Alyssum montanum* ecotypes under multi-metal stress. J. Plant Physiol..

[27] Wellburn AR (1994). The spectral determination of chlorophylls *a* and *b*, as well as total carotenoids, using various solvents with spectrophotometers of different resolution. J. Plant Physiol..

[28] Jiang HX, Chen LS, Zheng JG, Han S, Tang N (2008). Aluminium-induced effects on photosystem II photochemistry in *Citrus* leaves assessed by the chlorophyll a fluorescence transient. Tree Physiol..

[29] Kalaji HM, Bosa K, Kościelniak J, Żuk-Gołaszewska K (2011). Effects of salt stress on photosystem II efficiency and CO_2_ assimilation of two Syrian barley landraces. Environ. Exp. Bot..

[30] Hodgins, D.S. Yeast phenylalanine ammonia-lyase. Purification, properties, and the identification of catalytically essential dehydroalanine. *J. Biol. Chem.* **246(9)**, 2977–85 (1971). 5102931

[31] Fritz, R.R., Hodgins, D.S. & Abell, C.W. Phenylalanine ammonia-lyase. Induction and purification from yeast and clearance in mammals. *J. Biol. Chem.* **251(15)**, 4646–50 (1976). 985816

[32] Hura T, Dziurka M, Hura K, Ostrowska A, Dziurka K (2016). Different allocation of carbohydrates and phenolics in dehydrated leaves of triticale. J. Plant Physiol..

[33] Gołębiowska-Pikania G, Dziurka M, Wąsek I, Wajdzik K, Dyda M, Wędzony M (2019). Changes in phenolic acid abundance involved in low temperature and *Microdochium nivale* (Samuels and Hallett) cross-tolerance in winter triticale (× *Triticosecale* Wittmack). Acta Physiol. Plant..

[34] Dziurka M, Janeczko A, Juhász C, Gullner G, Oklestková J, Novák O (2016). Local and systemic hormonal responses in pepper leaves during compatible and incompatible pepper-tobamovirus interactions. Plant Physiol. Biochem..

[35] Hura T, Dziurka M, Hura K, Ostrowska A, Dziurka K, Gadzinowska J (2017). Wheat and rye genome confer specific phytohormone profile features and interplay under water stress in two phenotypes of triticale. Plant Physiol. Biochem..

[36] Płażek A, Dubert F, Kopeć P, Dziurka M, Kalandyk A, Pastuszak J (2018). Long-term effects of cold on growth, development and yield of narrow-leaf lupine may be alleviated by seed hydropriming or butenolide. Int. J. Mol. Sci..

[37] Sengar RS, Gautam M, Sengar RS, Garg SK, Senger K, Chaudhary R (2008). Lead stress effects on physiobiochemical activities of higher plants. Rev. Environ. Contam. Toxicol..

[38] Piwowarczyk, B., Tokarz, K., Muszyńska, E., Makowski, W., Jędrzejczyk, R., Gajewski, Z. et al. The acclimatization strategies of kidney vetch (*Anthyllis. vulneraria* L.) to Pb toxicity. *Environ. Sci. Pollut. Res.* **25**, 19739–19752 (2018). 10.1007/s11356-018-2197-6 PMC6061510 29736650

[39] Xiong Z, Zhao F, Li M (2006). Lead toxicity in *Brassica pekinensis* Rupr.: Effect on nitrate assimilation and growth. Environ. Toxicol..

[40] Khan MM, Islam E, Irem S (2018). Pb-induced phytotoxicity in para grass (*Brachiaria mutica*) and Castorbean (*Ricinus communis* L.): Antioxidant and ultrastructural studies. Chemosphere.

[41] Paunov M, Koleva L, Vassilev A, Vangronsveld J, Goltsev V (2018). Effects of different metals on photosynthesis: Cadmium and zinc affect chlorophyll fluorescence in durum wheat. Int. J. Mol. Sci..

[42] Szopiński M, Sitko K, Gieroń Ż, Rusinowski S, Corso M, Hermans C (2019). Toxic Effects of Cd and Zn on the photosynthetic apparatus of the *Arabidopsis halleri* and *Arabidopsis arenosa* pseudo-metallophytes. Front. Plant Sci..

[43] Chen W, Yang X, He Z, Feng Y, Hu F (2008). Differential changes in photosynthetic capacity, 77 K chlorophyll fluorescence and chloroplast ultrastructure between Zn-efficient and Zn-inefficient rice genotypes (*Oryza sativa*) under low zinc stress. Physiol. Plant..

[44] De Las Rivas, J. & Barber, J. Analysis of the structure of the PsbO protein and its implications. *Photosynth. Res.* **81**(3), 329–343 (2004). 10.1023/B:PRES.0000036889.44048.e4 16034536

[45] Kalaji HM, Jajoo A, Oukarroum A, Brestic M, Zivcak M, Samborska IA (2016). Chlorophyll a fluorescence as a tool to monitor physiological status of plants under abiotic stress conditions. Acta Physiol. Plant..

[46] Strasser, R.J., Tsimilli-Michael, M. & Srivastava, A. Analysis of the chlorophyll a fluorescence transient in *Chlorophyll a Fluorescence* (eds. Papageorgiou, C., Govindjee) 321–362 (Springer, Netherlands, 2004).

[47] Kováčik J, Klejdus B (2008). Dynamics of phenolic acids and lignin accumulation in metal-treated *Matricaria chamomilla* roots. Plant Cell Rep..

[48] Kováčik J, Dresler S, Wójciak-Kosior M, Babula P (2020). Uptake andphytotoxicity of lead are affected by nitrate nutrition and phenolic metabolism. Environ. Exp. Bot..

[49] Barros J, Escamilla-Trevino L, Song L, Rao X, Serrani-Yarce JC, Palacios MD (2019). 4-Coumarate 3-hydroxylase in the lignin biosynthesis pathway is a cytosolic ascorbate peroxidase. Nat. Commun..

[50] Niggeweg R, Michael AJ, Martin C (2004). Engineering plants with increased levels of the antioxidant chlorogenic acid. Nat. Biotechnol..

[51] Chen S, Lin R, Lu H, Wang Q, Yang J, Liu J (2020). Effects of phenolic acids on free radical scavenging and heavy metal bioavailability in *Kandelia obovata* under cadmium and zinc stress. Chemosphere.

[52] Kováčik J, Klejdus B, Hedbavny J, Zoń J (2011). Significance of phenols in cadmium and nickel uptake. J. Plant Physiol..

[53] Irtelli B, Navari-Izzo F (2006). Influence of sodium nitrilotriacetate (NTA) and citric acid on phenolic and organic acids in *Brassica juncea* grown in excess of cadmium. Chemosphere.

[54] Dresler, S., Wójciak-Kasior, M., Sowa, I., Stanisławski, G., Bany, I. & Wójcik, M. Effect of short-term Zn/Pb or long-term multi-metal stress on physiological and morphological parameters of metallicolous and nonmetallicolous *Echium vulgare* L. populations. *Plant Physiol. Biochem*. **115**, 380–389 (2017). 10.1016/j.plaphy.2017.04.016 28432977

[55] Kovács V, Gondor OG, Szalai G, Darkó E, Majláth I, Janda T (2014). Synthesis and role of salicylic acid in wheat varieties with different levels of cadmium tolerance. J. Hazard. Mat..

[56] Choudhary SP, Bhardwaj R, Gupta BD, Dutt P, Gupta RK, Kanwar M (2010). Changes induced by Cu^2+^ and Cr^6+^ metal stress in polyamines, auxins, abscisic acid titers and antioxidative enzymes activities of radish seedlings. Braz. J. Plant Physiol..

[57] Kim Y-H, Khan AL, Kim D-H, Lee S-Y, Kim K-M, Waqas M (2014). Silicon mitigates heavy metal stress by regulating P-type heavy metal ATPases, *Oryza sativa* low silicon genes, and endogenous phytohormones. BMC Plant Biol..

[58] De Ollas C, Dodd IC (2016). Physiological impacts of ABA–JA interactions under water-limitation. Plant Mol. Biol..

[59] Shan C, Liang Z (2010). Jasmonic acid regulates ascorbate and glutathione metabolism in *Agropyron cristatum* leaves under water stress. Plant Sci..

[60] Sirhindi G, Mir MA, Abd-Allah EF, Ahmad P, Gucel S (2016). Jasmonic acid modulates the physio-biochemical attributes, antioxidant enzyme activity, and gene expression in *Glycine max* under nickel toxicity. Front. Plant. Sci..

