# Supplementary material for: Photosynthetic apparatus efficiency, phenolic acid profiling and pattern of chosen phytohormones in pseudometallophyte Alyssum montanum
Source: Sci Rep. 2021 Feb 18;11:4135. doi: 10.1038/s41598-021-83695-y (PMC7892566; doi:10.1038/s41598-021-83695-y)
Supplement: Supplementary file 1 — Supplementary Information. [file 41598_2021_83695_MOESM1_ESM.docx]

**Photosynthetic apparatus efficiency, phenolic acid profiling and pattern of chosen phytohormones in pseudometallophyte *Alyssum montanum***

Ewa Muszyńska, Krzysztof Tokarz, Michał Dziurka, Mateusz Labudda, Kinga Dziurka, Barbara Tokarz

| 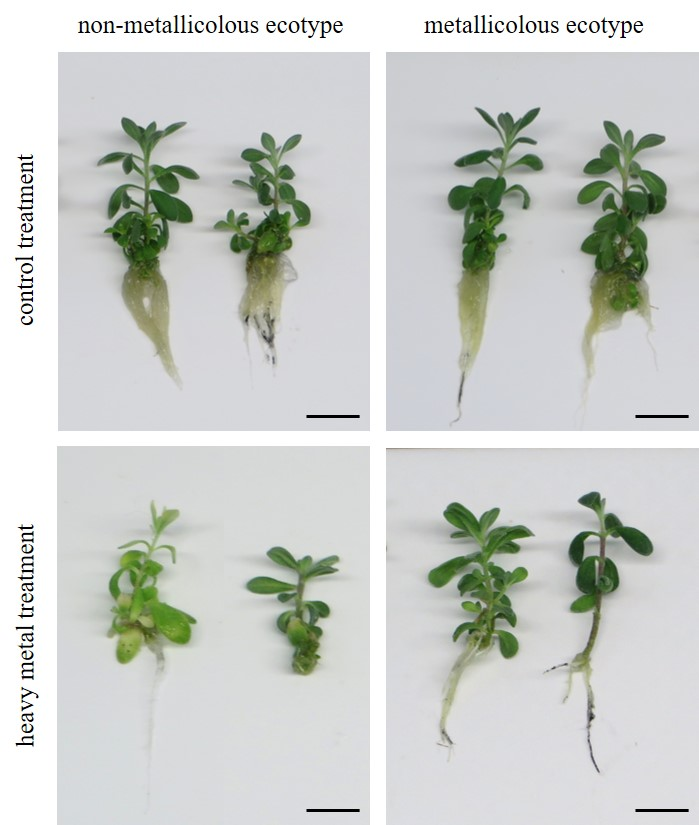 |
| --- |
| **Supplementary Figure S1.** Morphology of tested specimens cultivated for 8 weeks on medium without zinc, lead and cadmium (control treatment) as well as on medium enriched with these metallic elements (heavy metal treatment). Bars = 10 mm. |

| 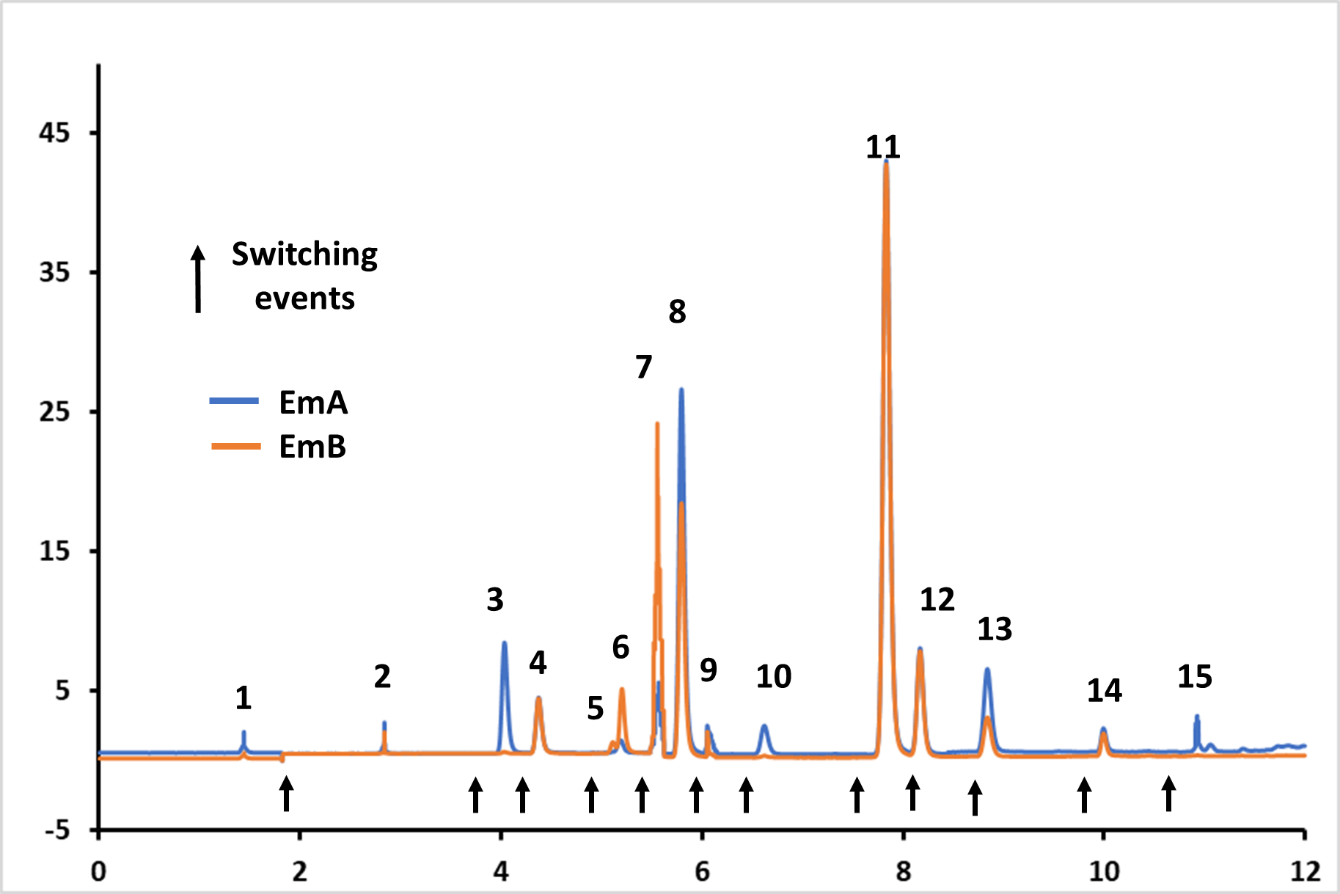 |
| --- |
| **LU vs. Time (min)** |
| 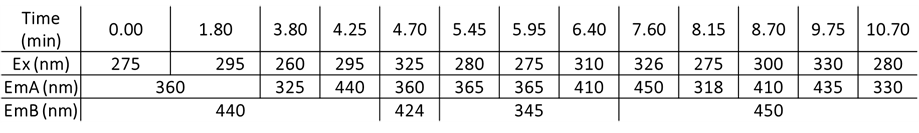 |
| **Supplementary Figure** **S2.** Chromatogram of pure phenolic acids standards mixture with time programed wavelength switching for increased selectivity through optimum wavelength settings for compounds of interest at their retention region. Two excitation/emission wavelengths settings are used (blue and red traces). Monitored compounds according retention times: gallic (1), protocatechuic (2) p-hydroxybenzoic (3), gentisic (4), caffeic (5), vanillic (6), chlorogenic (7), homovanilic (8), syringic (9), p-coumaric (10), ferulic (11), sinapic (12), salicylic (13), rosmarinic (14) and cinnamic (15) acids. |

| 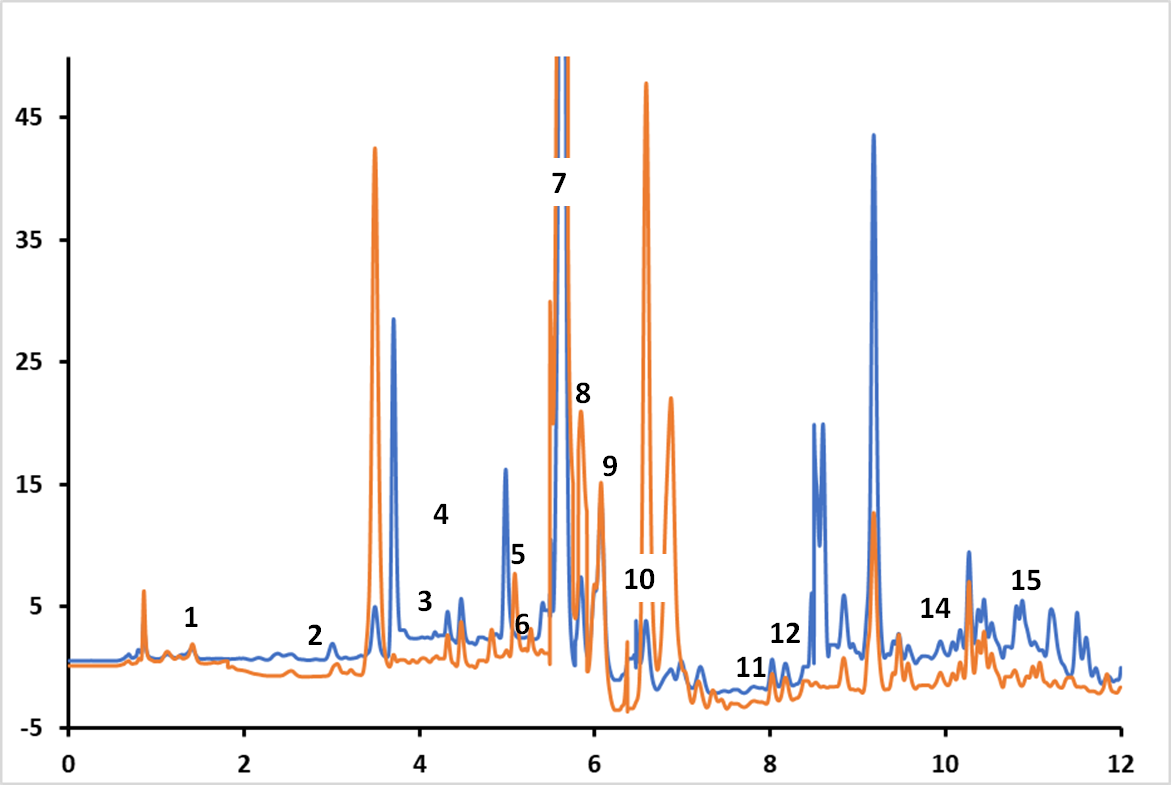 |
| --- |
| **LU vs. Time (min)** |
| **Supplementary Figure** **S3**. Representative chromatogram of non-metallicolous ecotype plant sample. Two excitation/emission wavelengths settings are used (blue and red traces). Monitored compounds according retention times: gallic (1), protocatechuic (2) p-hydroxybenzoic (3), gentisic (4), caffeic (5), vanillic (6), chlorogenic (7), homovanilic (8), syringic (9), p-coumaric (10), ferulic (11), sinapic (12), rosmarinic (14) and cinnamic (15) acids. |

| 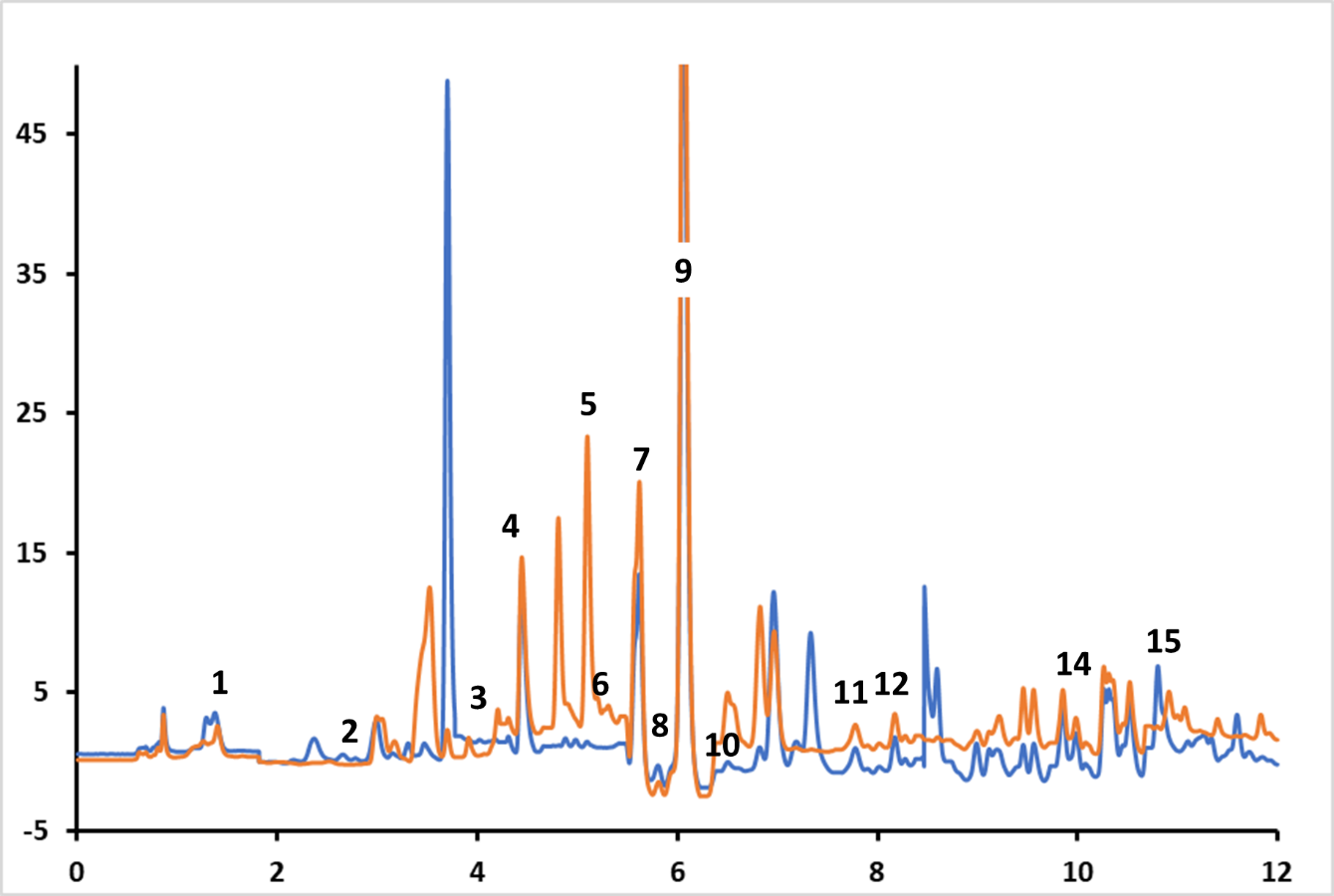 |
| --- |
| **LU vs. Time (min)** |
| **Supplementary Figure S4.** Representative chromatogram of metallicolous ecotype plant sample. Two excitation/emission wavelengths settings are used (blue and red traces). Monitored compounds according retention times: gallic (1), protocatechuic (2) p-hydroxybenzoic (3), gentisic (4), caffeic (5), vanillic (6), chlorogenic (7), homovanilic (8), syringic (9), p-coumaric (10), ferulic (11), sinapic (12), rosmarinic (14) and cinnamic (15) acids. |

| **A** | 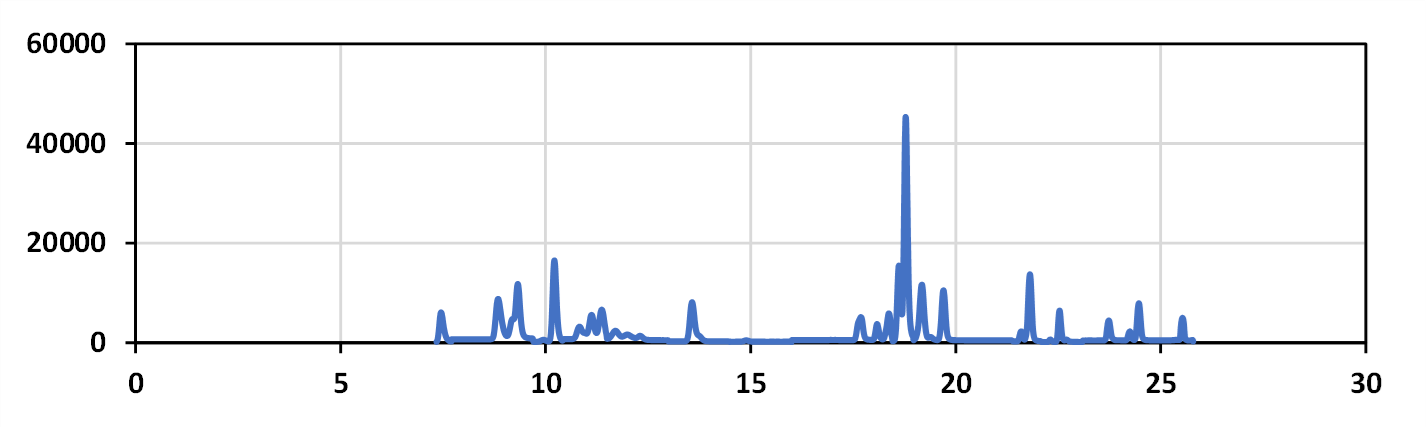 |
| --- | --- |
| **B** | 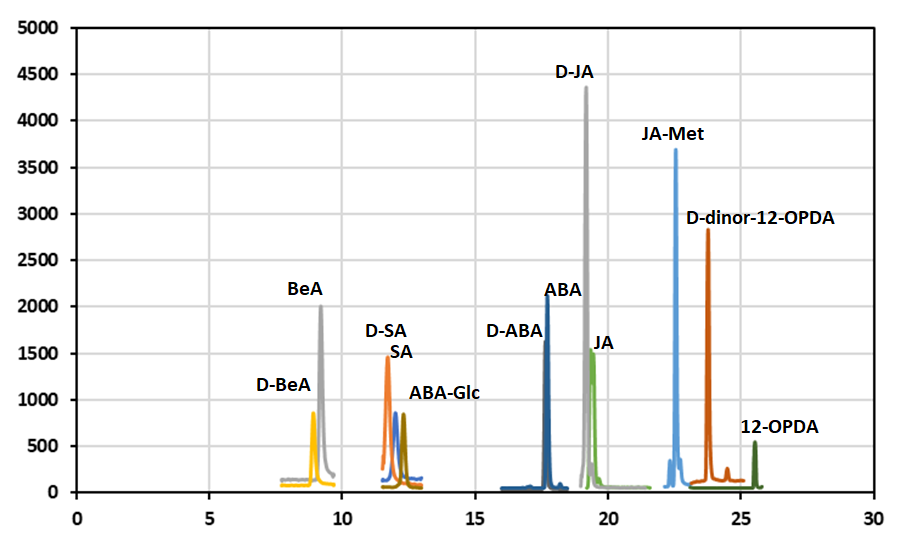 |
|  | **Counts vs. Acquisition time (min)** |
| **C** | \| Compound \|  \| Type of ion \| Quantifier transition  (precursor/product ions) \| Fragmentor voltage (V) \| Collision energy (V) \| Retention Time (min) \| \| --- \| --- \| --- \| --- \| --- \| --- \| --- \| \| D-SA \| ISTD \| [M+H]^+^ \| 143.2/125.2 \| 80 \| 14 \| 11.60 \| \| SA \|  \| [M+H]^+^ \| 139.2/121.2 \| 80 \| 14 \| 11.93 \| \| D-BeA \| ISTD \| [M+H]^+^ \| 128.1/84.2 \| 60 \| 14 \| 8.48 \| \| BeA \|  \| [M+H]^+^ \| 124.1/80.0 \| 60 \| 14 \| 9.05 \| \| D-ABA \| ISTD \| [M-H_2_O+H]^+^ \| 253.4/191.3 \| 80 \| 14 \| 17.59 \| \| ABA \|  \| [M-H_2_O+H]^+^ \| 247.4/187.2 \| 80 \| 14 \| 17.67 \| \| ABA-Glc \|  \| [M-H_2_O+H]^+^ \| 409.2/247.1 \| 104 \| 14 \| 12.24 \| \| D-JA \| ISTD \| [M+H]^+^ \| 216.3/153.2 \| 80 \| 5 \| 19.14 \| \| JA \|  \| [M+H]^+^ \| 211.3/151.2 \| 80 \| 14 \| 19.20 \| \| MeJA \|  \| [M+H]^+^ \| 225.3/151.2 \| 58 \| 5 \| 22.51 \| \| D-dinor-12-OPDA \| ISTD \| [M+H]^+^ \| 270.3/252.2 \| 84 \| 5 \| 23.72 \| \| 12-OPDA \|  \| [M+H]^+^ \| 293.3/275.2 \| 68 \| 9 \| 25.54 \| |
| **Supplementary Figure** **S5.** UHPLC-MS/MS analysis of phytohormones pure standards mixture. A) Total ion current (TIC) chromatogram of analyzed sample. B) Multiple reactions monitoring (MRM) transitions chromatogram for the analyzed plant hormones and other compounds. C) MRM parameters at: positive ion mode (+ESI), capillary voltage 4 kV, gas temperature 350 °C, gas flow 12 l/min and nebulizer pressure 35 psi. MassHunter software was used to control the LC–MS/MS system and in data analysis. For MRM parameters optimization MassHunter Optimizer was used. | |

| **A** | 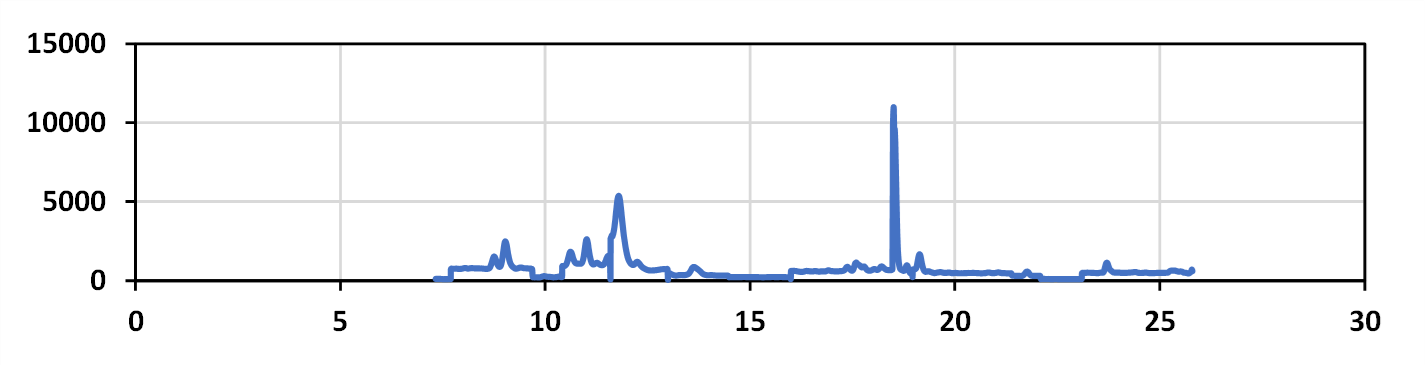 |
| --- | --- |
| **B** | 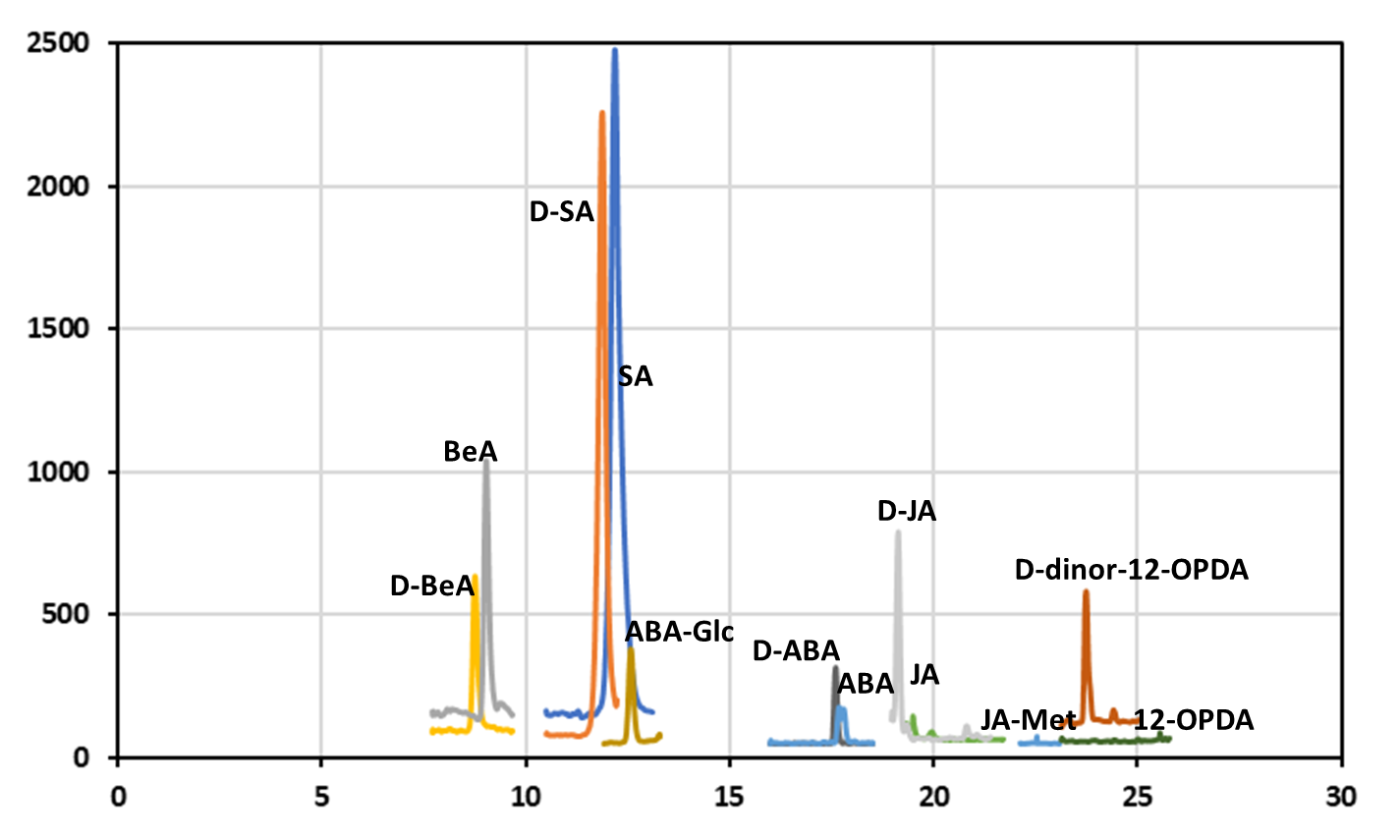 |
|  | **Counts vs. Acquisition time (min)** |
| **Supplementary Figure** **S6.** UHPLC-MS/MS analysis of representative non-metallicolous ecotype plant sample. A) Total ion current (TIC) chromatogram of analyzed sample. B) Multiple reactions monitoring (MRM) transitions chromatogram for the analyzed plant hormones and other compounds. | |

| **A** | 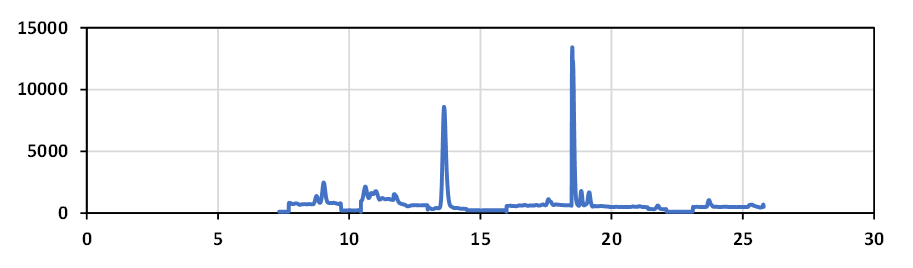 |
| --- | --- |
| **B** | 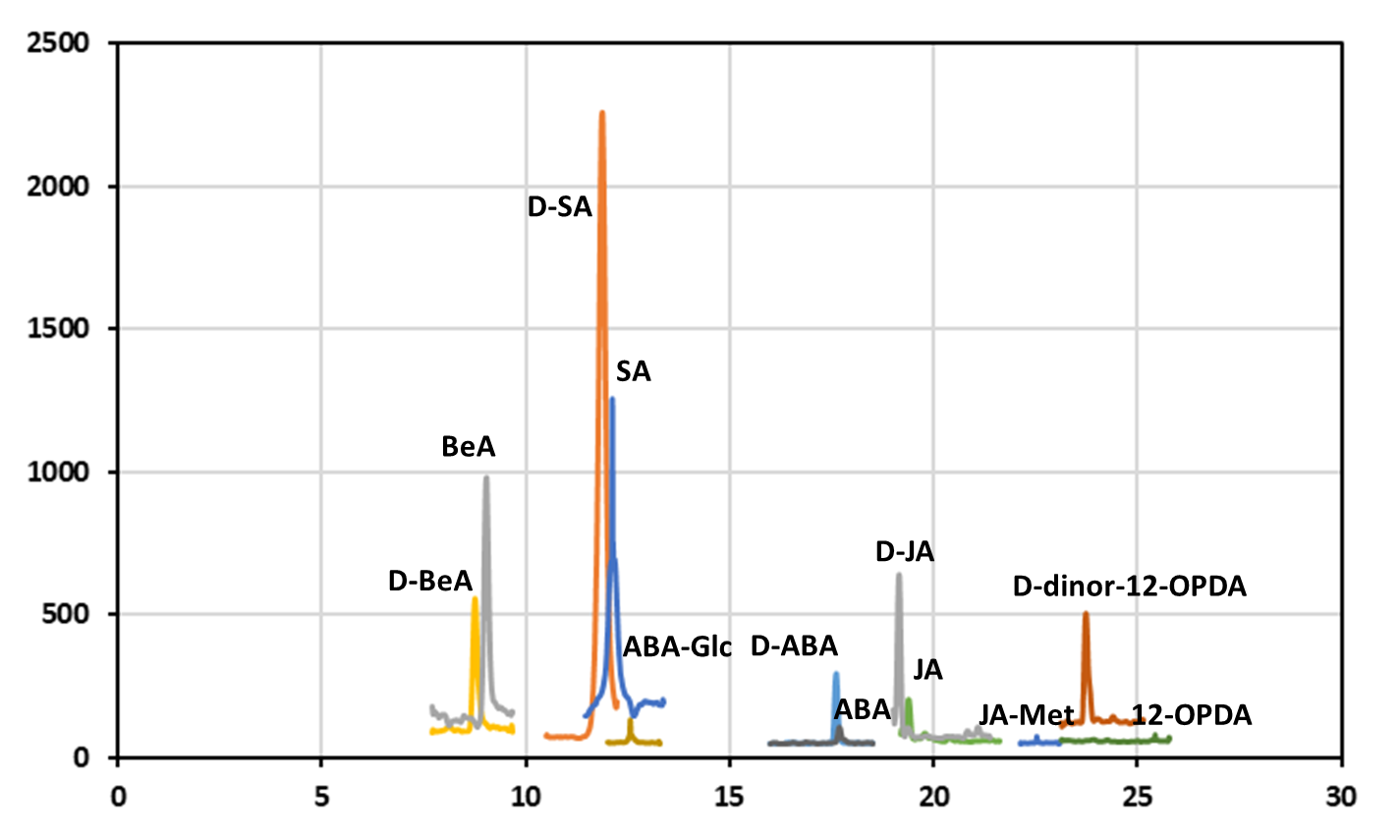 |
|  | **Counts vs. Acquisition time (min)** |
| **Supplementary Figure** **S7.** UHPLC-MS/MS analysis of representative metallicolous ecotype plant sample. A) Total ion current (TIC) chromatogram of analyzed sample. B) Multiple reactions monitoring (MRM) transitions chromatogram for the analyzed plant hormones and other compounds. | |
